# Autoinflammatory encephalopathy due to PTP1B haploinsufficiency: a case series

**DOI:** 10.1016/S1474-4422(24)00526-X

**Published:** 2025-03-01

**Authors:** Gaofeng Zhu, Blaise Didry-Barca, Luis Seabra, Gillian I Rice, Carolina Uggenti, Moncef Touimy, Mathieu P Rodero, Rolando Hernandez Trapero, Vincent Bondet, Darragh Duffy, Philippe Gautier, Katie Livingstone, Fraser JH Sutherland, Pierre Lebon, Mélanie Parisot, Christine Bole-Feysot, Cécile Masson, Nicolas Cagnard, Patrick Nitschké, Glenn Anderson, Birgit Assmann, Magalie Barth, Odile Boespflug-Tanguy, Felice D’Arco, Imen Dorboz, Thomas Giese, Yael Hacohen, Miroslava Hancarova, Marie Husson, Anne Lepine, Ming Lim, Maria Margherita Mancardi, Isabelle Melki, David Neubauer, Mario Sa, Zdenek Sedlacek, Angelika Seitz, Mika Shapiro Rottman, Sylvia Sanquer, Rachel Straussberg, Markéta Vlčková, Frédéric Villéga, Matias Wagner, Ayelet Zerem, Joseph A Marsh, Frémond Marie-Louise, Marios Kaliakatsos, Yanick J Crow, Marie-Thérèse El-Daher, Alice Lepelley

**Affiliations:** 1 MRC Human Genetics Unit, Institute of Genetics and Cancer, University of Edinburgh, Edinburgh, United Kingdom; 2 Laboratory of Neurogenetics and Neuroinflammation, Imagine Institute, INSERM UMR1163, Université Paris Cité, Paris, France; 3 Division of Evolution and Genomic Sciences, School of Biological Sciences, Faculty of Biology, Medicine and Health, University of Manchester, Manchester Academic Health Science Centre, Manchester, United Kingdom; 4 Translational Immunology Unit, Institut Pasteur, Université de Paris Cité, F75015 Paris, France; 5 Medical School, Paris City University, Paris, France; 6 Genomics Core Facility, Institut Imagine-Structure Fédérative de Recherche Necker, INSERM U1163 et INSERM US24/ CNRS UAR3633, Paris Cite University, Université Paris Cité, Paris, France; 7 Bioinformatics Platform, Institut Imagine-Structure Fédérative de Recherche Necker, INSERM U1163 et INSERM US24/ CNRS UMS3633, Université Paris Cité, Paris, France; 8 Department of Histopathology, Camelia Botnar Laboratories, Great Ormond Street Hospital for Children, London, United Kingdom; 9 Heidelberg University, Medical Faculty Heidelberg, Center for Pediatric and Adolescent Medicine Department I, Division of Pediatric Neurology and Metabolic Medicine, Heidelberg, Germany; 10 Service de génétique, Centre Hospitalier Universitaire d’Angers, Angers, France; 11 APHP centre de référence LEUKOFRANCE service de Neuropediatrie Hopital Robert Debre, Paris, France; 12 Universite Paris Cité NeuroDiderot UMR INSERM 1141, Hopital Robert Debre, Paris, France; 13 Department of Radiology, Great Ormond Street Hospital for Children, London, United Kingdom; 14 Institute of Immunology and German Center for Infection Research (DZIF), partner site Heidelberg, Heidelberg University Hospital, Heidelberg, Germany; 15 Queen Square MS Centre, UCL Queen Square Institute of Neurology, Faculty of Brain Sciences, University College London, London, United Kingdom; 16 Department of Neurology, Great Ormond Street Hospital for Children, London, United Kingdom; 17 Department of Biology and Medical Genetics, Charles University Second Faculty of Medicine and University Hospital Motol, Prague, Czech Republic; 18 Unité de neurologie de l’enfant et de l’adolescent, CHU Pellegrin, Bordeaux, France; 19 Service de Neuropédiatrie, Hôpital de la Timone Enfants, Marseille, France; 20 Evelina London Children’s Hospital, Guy’s and St Thomas’ NHS Foundation Trust, London, United Kingdom; 21 Department Women and Children’s Health, School of Life Course Sciences (SoLCS), King’s College London, London, United Kingdom; 22 Unit of Child Neuropsychiatry, EpiCARE Network, IRCCS Giannina Gaslini, Genova, Italy; 23 Department of General Pediatrics, Armand Trousseau Hospital, Assistance Publique-Hôpitaux de Paris (AP-HP), Sorbonne Université, Paris, France; 24 Department of Child, Adolescent & Developmental Neurology, University Children’s Hospital, Ljubljana, Slovenia; 25 Department of Paediatric Neurology, Oxford University Hospitals NHS Foundation Trust, Oxford, United Kingdom; 26 Department of Neuroradiology, Heidelberg University Hospital, Heidelberg, Germany; 27 Department of diagnostic imaging, Rambam Health Care Campus, Faculty of Medicine, Technion, Haifa, Israel; 28 Biochemistry, Metabolomics and Proteomics Department, Necker Hospital, AP-HP Centre, Université Paris Cité, Paris, France; 29 Institute of Pediatric Neurology, Schneider Children’s Medical Center of Israel, Petach Tikva, Israel; 30 Faculty of Medical and Health Sciences, Tel Aviv University, Tel Aviv, Israel; 31 Institute of Human Genetics, Klinikum rechts der Isar, School of Medicine, Technical University of Munich, Munich, Germany; 32 Institute for Neurogenomics, Helmholtz Zentrum München, Neuherberg, Germany; 33 Division of Pediatric Neurology, Developmental Neurology and Social Pediatrics, Dr. von Hauner Children’s Hospital, Munich, Germany; 34 Pediatric Neurology Institute, Dana-Dwek Children’s Hospital, Tel Aviv Sourasky Medical Center, Faculty of Medicine, Tel Aviv University, Tel Aviv, Israel; 35 Reference center for inflammatory Rheumatism, AutoImmune diseases and Systemic interferonopathies in childrEn (RAISE), Paris, France; 36 Department of Paediatric Hematology-Immunology and Rheumatology, Necker-Enfants Malades Hospital, Assistance publique–hôpitaux de Paris (AP-HP), Paris, France; 37 Department of Neuroscience, Institute of Child Health, University College London, London, United Kingdom

## Abstract

**Background:**

Through the agnostic screening of patients with uncharacterised disease phenotypes for an upregulation of type I interferon (IFN) signalling, we identified a cohort of individuals heterozygous for mutations in *PTPN1*, encoding the protein-tyrosine phosphatase PTP1B. We aimed to describe the clinical phenotype and molecular and cellular pathology of this new disease.

**Methods:**

We ascertained patients, clinical and neuroradiological data through paediatric neurology and clinical genetics colleagues across Europe and Israel. Variants in *PTPN1* were identified by exome and directed Sanger sequencing. The expression of IFN stimulated genes (ISGs) was determined by qPCR or NanoString technology. Experiments to assess RNA and protein expression, and to investigate type I IFN signalling, were undertaken in patient fibroblasts, hTERT immortalised BJ-5ta fibroblasts and RPE-1 cells using CRISPR/Cas9 editing and standard cell biology techniques.

**Findings:**

We identified ten novel or very rare variants (frequency on gnomAD v4.1.0 < 1.25 x 10^-6^) in *PTPN1* in 12 patients from 11 families recruited between 20^th^ December 2013 and 11^th^ January 2023. Six variants were predicted as STOP mutations, two involved canonical splice site nucleotides, and two were missense substitutions. In three patients the variant occurred de novo, while in nine the variants were inherited from an asymptomatic parent. The clinical phenotype was characterised by the subacute onset (age range 1 - 8 years) of loss of skills in the absence of seizures after initially normal development, leading to spastic-dystonia and bulbar involvement. Neuroimaging variably demonstrated cerebral atrophy (sometimes unilateral initially) and/or high T2 white matter signal. Cerebrospinal fluid (CSF) neopterin was elevated in all ten patients tested, and all probands demonstrated an upregulation of ISGs in whole blood. While clinical stabilisation and neuroradiological improvement was seen in both treated and untreated patients, in six of eight treated cases high dose corticosteroids were judged clinically to result in an improvement in neurological status. Of the four asymptomatic parents tested, IFN signalling in blood was normal (three) or minimally elevated (one). Analysis of patient blood and fibroblasts showed that tested *PTPN1* variants led to reduced levels of *PTPN1* mRNA and/or PTP1B protein, and in vitro assays demonstrated that loss of PTP1B function is associated with impaired negative regulation of type I IFN signalling.

**Interpretation:**

PTP1B haploinsufficiency causes a type I IFN driven autoinflammatory encephalopathy. Notably, some patients demonstrated stabilisation, and even recovery, of neurological function in the absence of treatment, while in others the disease appeared to be responsive to immune suppression. Prospective studies are needed to investigate the safety and efficacy of specific immune suppression approaches in this disease population.

**Funding:**

UK Medical Research Council (MRC), European Research Council (ERC) and Agence nationale de la recherche (ANR).

## Introduction

Reversible protein phosphorylation is a major cellular regulatory mechanism, with members of the protein tyrosine phosphatase (PTP) family acting to maintain homeostatic protein tyrosine kinase signalling by removing phosphate moieties from phosphorylated substrates.^1^ Phosphatases are widely expressed in the immune system, serving as key regulators of signalling in multiple types of immune cell.^2^

Protein-tyrosine phosphatase 1B (PTP1B), encoded by *PTPN1*, is a highly evolutionarily conserved, ubiquitously expressed, cytoplasmic non-receptor-type PTP.^3^ *Ptpn1* knock-out (KO) mice are both viable and healthy, demonstrating enhanced insulin sensitivity and resistance to metabolic syndrome.^4^ However, heterozygous somatic null variants in *PTPN1* act as driver mutations in Hodgkin lymphoma and primary mediastinal B cell lymphoma, associated with reduced phosphatase activity and increased phosphorylation of Janus kinase-signal transducer and activator of transcription (JAK-STAT) pathway components, indicative of loss of a negative brake on oncogenic JAK-STAT activation.^5^ Of note, PTP1B has been implicated as a negative regulator of type I interferon (IFN) signalling,^6^ through a variety of proposed mechanisms including tyrosine kinase 2 (TYK2) dephosphorylation and degradation of stimulator of IFN genes (STING).^7,8^

The closest paralogue to PTP1B is PTPN2 (also known as TC-PTP) encoded by *PTPN2*, with shared identity in their catalytic and substrate recognition domains manifesting as a common activity for some substrates.^9^ Notably, human germline heterozygous and biallelic variants in *PTPN2*, leading to haploinsufficiency or loss-of-function (LOF) of the encoded protein, have recently been associated with autoimmune enteropathy,^10,11^ immunodeficiency,^12^ and systemic autoimmunity including systemic lupus erythematosus and cytopenias.^12,13^

We undertook the current study in order to characterise and report the neurological disease associated with germline autosomal dominant LOF mutations in *PTPN1*.

## Role of the funding source

The funders of the study had no role in study design, data collection, data analyses, data interpretation, or writing of the report.

## Methods

### Study design

Patients were ascertained clinically through paediatric neurologists and clinical geneticists internationally. Research testing was undertaken in the MRC Human Genetics Unit, Institute of Genetics and Cancer, University of Edinburgh, and in the Imagine Institute, Paris. The clinical features of AGS1036, AGS1312 and AGS1421 were previously described by Sa et al. (as Cases 2, 3 and 1, respectively, in that report).^14^

### Participants

Patient clinical and neuroradiological data were collated, including clinical investigations undertaken as part of routine hospital care. Samples for research testing were obtained from probands and parents with written informed consent.

### Genetic studies

DNA was extracted from whole blood. Exome sequencing was performed local to patients. Following the identification of three individuals with de novo mutations in *PTPN1*, targeted Sanger sequencing was undertaken in other patients with a similar phenotype. Identified variants were assessed for their frequency in the Genome Aggregation Database (gnomAD version 4.1.0), and for their predicted effect on the encoded protein. The splicing module of Alamut® Visual was used to predict the effect of variants on splicing, and in silico programs (Combined Annotation Dependent Depletion, Sorting Intolerant From Tolerant, and Polymorphism Phenotyping version 2) and protein modelling (using ChimeraX and FoldX) were employed to predict the effect of non-synonymous missense variants on PTP1B. Inheritance was determined where parental DNA was available.

### IFN status

The expression of IFN stimulated genes (ISGs) in blood was assessed either by qPCR or using NanoString technology.^15,16^ IFN-alpha protein levels and IFN-alpha activity were measured, respectively, by digital ELISA or cytopathic protection assay.^17,18^

### In vitro experiments

Experiments to assess RNA and protein expression, and to investigate type I IFN signalling, in the context of wild-type (WT) or mutant PTPN1, were undertaken in patient fibroblasts, BJ-5ta hTERT immortalised fibroblasts and hTERT RPE-1 cells using CRISPR-Cas9 gene editing and standard cell biology techniques.

Further details on the methods used in this study are given in the Supplementary file.

### Statistics

Data are presented as mean ± SEM (standard error of the mean). All statistical testing was undertaken in GraphPad Prism 10. For comparison between two groups, unpaired *t* test or ratio paired *t* test was chosen when appropriate (as indicated in figure legends). For comparison among three or more groups of the same categorial variable (i.e., genotype in this manuscript), One-way ANOVA was used. For comparison among three or more groups of two independent categorical variables (i.e., genotype and treatment in this manuscript), mixed-model two-way ANOVA was adopted. *p* values are annotated in each graph. For more details, see the Statistics section in the Supplementary file. For RNA sequencing analysis with DESeq2, the adjusted *p* values were obtained by the Wald test with Benjamini and Hochberg correction for multiple comparisons. In GSEA (gene set enrichment analysis), nominal *p* values were calculated using an empirical gene set-based permutation test, and FDR (false discovery rate) was adjusted for gene set size and multiple hypotheses testing. For more details see reference 9 in the Supplementary file.

## Results

**Figure 1:**
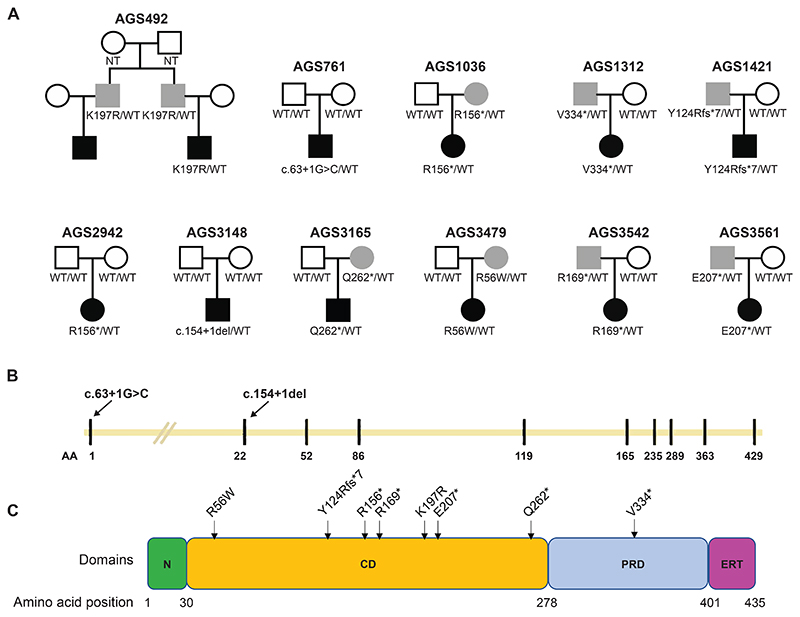
Genetic data (A) Family pedigrees where an affected individual carries a novel or very rare heterozygous non-synonymous substitution in *PTPN1*. Circles and squares indicate female and males respectively. Dark and light colouring denote, respectively, affected and clinically asymptomatic mutation-positive individuals. NT = not tested; WT = wild-type. (B) Cartoon of *PTPN1* locus with perpendicular blue lines indicating exons numbered above. Numbers below (1, 22, 52, etc) indicate the first amino acid (AA) position in the respective exon. The two splicing variants are depicted on the top with red arrows showing the relative position next to the exon. (C) Cartoon of the protein domains of PTP1B, with amino acid numbering below. STOP mutations and non-synonymous missense substitutions are indicated. N = N terminal domain; CD = catalytic domain; PRD = proline-rich domain; ERT = ER-targeting domain.

As part of an ongoing research strategy involving the agnostic screening of patients with uncharacterised disease phenotypes for an upregulation of type I IFN signalling, we identified a male child experiencing infantile-onset subacute loss of skills to carry a de novo c.63+1G>C canonical donor splice site in intron 1 of *PTPN1* (Table 1). Using GeneMatcher, we ascertained two further patients with de novo variants in *PTPN1*: a c.466C>T transition predicted to result in a STOP mutation (p.(Arg156*), R156*), and a c.154+1del canonical donor splice site variant in intron 2. Through international colleagues we then identified nine further patients (with two affected first cousins in family AGS492) heterozygous for either a STOP mutation (six probands) (p.(Tyr124Argfs*7), Y124Rfs*7; p.(Arg156*), R156*; p.(Arg169*), R169*; p.(Glu207*), E207*; p.(Gln262*), Q262*; p.(Val334*), V334*) or a rare missense substitution (two probands) (p.(Arg56Trp), R56W; p.(Lys197Arg), K197R) in PTP1B (Figure 1A-C).

Of the ten putative mutations (the identical R156* being seen in two families, and the K197R present in two affected first cousins from one family), six were predicted to introduce a premature stop codon, and two involved canonical splice site nucleotides likely to disrupt mRNA splicing and severely affect protein sequence. Further, while the two missense substitutions are predicted to have relatively mild effects on protein stability, they occur at evolutionarily conserved residues that have been described as playing important roles in the allosteric regulation of PTP1B function (Supplementary Figure 1A). *PTPN1* is a highly constrained gene with a pLI of 1, indicating strong negative selection against LOF variants. Consistent with this, of the variants recorded in our cohort, only the R56W and the R169* were present in gnomAD v4.1.0 (seen in the heterozygous state twice and once, respectively, out of > 1,600,000 alleles). While in three patients the variant appeared to have occurred de novo, in nine cases the variants were inherited from an asymptomatic parent (with two asymptomatic fathers to the two affected first cousins in family AGS492). It is of note that the R156* mutation was seen to occur de novo in one family (AGS2942), and to have been inherited from an asymptomatic mother in another (AGS1036) (Figure 1A). Of further note, the R156* and R56W mutations were previously reported as somatic driver mutations in, respectively, primary mediastinal B cell lymphoma and Hodgkin lymphoma.

The clinical phenotype of the 12 symptomatic cases was remarkable for the high degree of stereotypy observed (Table 2; Supplementary Table 1; Clinical histories in Supplemental data). All 12 symptomatic patients (100%) experienced subacute loss of skills (age range 15 months to 8 years) after initial normal development in 11 of 12 (92%) (one patient demonstrated mild motor delay i.e. did not walk independently until 21 months of age), with all 12 patients (100%) demonstrating weakness, spasticity (initially manifesting as a hemiparesis in seven patients (58%), and then becoming bilateral) +/- dystonia, and 11 of 12 (92%) exhibiting bulbar involvement (dysphagia and/or dysphasia) in the absence of seizures (although noting that one patient experienced a febrile seizure one month before the onset of loss of skills). In four of 12 patients, fever and raised liver enzymes were noted around the time of presentation.

**Figure 2:**
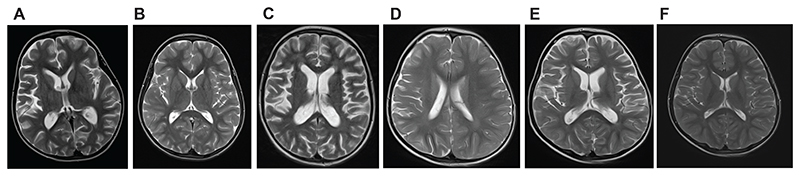
Representative axial T2 cerebral MRIs in affected patients. (A) AGS1036 at age 5 years, 5 months post symptom-onset, showing generalised volume loss more evident in the right cerebral hemisphere. (B) AGS1036 at age 8 years, 35 months post symptom-onset and 23 months post initiation of treatment, showing almost complete normalisation of the changes seen initially. (C) AGS2942 at age 9 years showing significant cortico-subcortical atrophy with passive enlargement of the ventricles and cerebral sulci, and some hypersignal of the deep white matter frontally. (D) AGS3542 at age 2 years 2 months showing right-sided hemi-atrophy and non-specific hypersignal of the deep white matter. (E) AGS3542 at age 3 years 8 months showing bilateral atrophy. (F) AGS3542 at age 7 years 8 months showing normalised brain volume without significant asymmetry and normalised white matter.

Cerebral atrophy (9/12 patients: 75%), initially unilateral in four cases (33%), was the major neuroradiological feature, with non-specific white matter changes seen in eight individuals (67%), and intracranial calcification reported in two patients (17%) (Figure 2). Four patients underwent brain biopsy, demonstrating non-specific inflammation in three (Supplementary Table 1).

All 12 patients (100%) experienced subsequent clinical stabilisation, with all (100%) alive at last contact (age range 3 − 20 years). While the majority of children were left with severe to profound motor and communication difficulties (GMCFS and/or CFCS ≥ IV in seven patients (58%)), in two cases (17%) there was a return to (AGS2942), or close to (AGS1036), pre-disease levels of function. In eight cases (67%) brain imaging showed complete, or near complete, resolution of the cerebral atrophy and/or white matter disease seen in the initial stages of the disease. Eight of twelve patients (67%) received immunosuppression. Clinical stabilisation and neuroradiological improvement were seen in both treated and untreated patients. However, in six of eight treated cases it was judged clinically that the improvement in neurological status was likely related to immune suppression with high dose corticosteroids, with one patient (AGS3542) showing an apparent steroid dependency over more than two years (Table 3; clinical histories in Supplemental data; pre- and post-treatment videos of AGS1036, AGS1312 and AGS1421 available as, respectively, video 2, video 3 and video 1 at: https://www.sciencedirect.com/science/article/pii/S1090379817318317?via%3Dihub#ec1).

Of possible note, clinical improvement following treatment was associated with normalisation of ISG expression in whole blood in two of these patients (AGS1036 and AGS1421) (Supplementary Figure 1B).

The nine asymptomatic carriers, aged between 34 and 52 years, did not report any significant past medical history.

**Figure 3:**
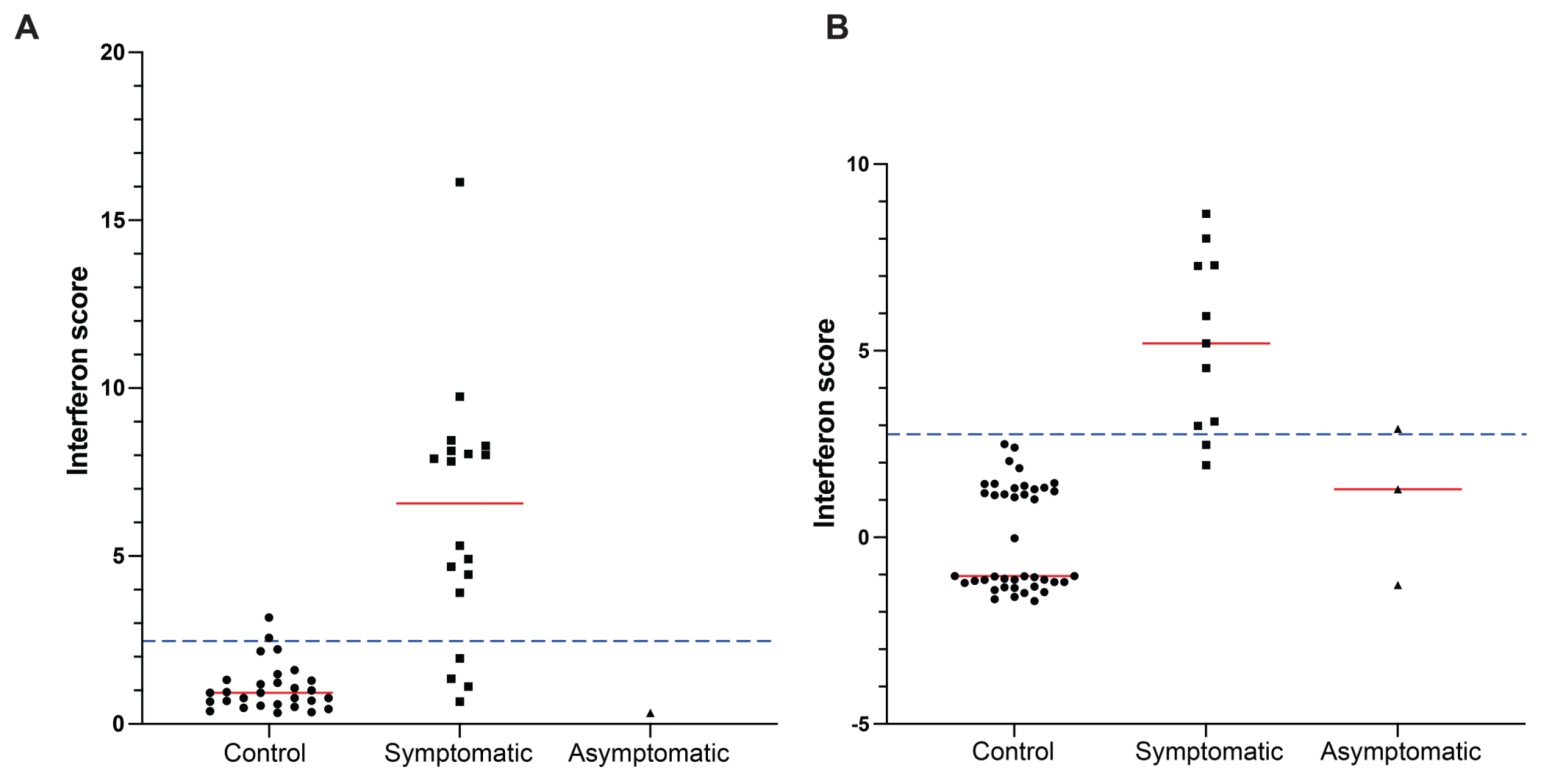
Interferon scores in patients and asymptomatic parents Interferon (IFN) scores (median fold expression of a panel of IFN stimulated genes (ISGs)) recorded in symptomatic patients and their asymptomatic parents carrying a mutation in *PTPN1*. In families AGS492 – AGS1421 IFN scores were derived using a six ISG panel measured by qPCR (A),^15^ while in families AGS2942 – AGS3561 a 24 ISG panel was generated using NanoString technology (B).^16^ The upper range of normal is calculated as +2 SD above the mean of the control group, as indicated by the dotted lines: qPCR = 2.466; NanoString = 2.758. Red lines indicate the median values for the respective groups.

Cerebrospinal fluid (CSF) levels of neopterin, a marker of central nervous system inflammation, consistently raised in type I interferonopathies involving the neurological system, were elevated in all ten (100%) patients where tested (range 2 – 45 multiples of laboratory-specific upper limits of normal) (Supplementary Table 2). Further, all 12 patients (100%) demonstrated evidence of enhanced IFN signalling, with an upregulation of ISGs in whole blood on at least one occasion. Overall, 24 of 29 such results were abnormal (Figure 3; Supplementary Table 2), with serial testing revealing a fall of ISGs over time consistent with the observed clinical stabilisation. We also recorded a marked increase of IFN-alpha protein in the CSF and serum in the one patient tested, and increased IFN-alpha activity in the CSF of two patients on each occasion tested (Supplementary Table 2). Additionally, tubuloreticular inclusions, subcellular structures located within the cisternae of the endoplasmic reticulum, a marker of enhanced IFN signalling, were observed in the brain biopsy of AGS1036 (Supplementary Figure 1C). Of four asymptomatic mutated parents tested, IFN signalling was normal in three, and minimally elevated in one (clinical histories in Supplemental data).

**Figure 4:**
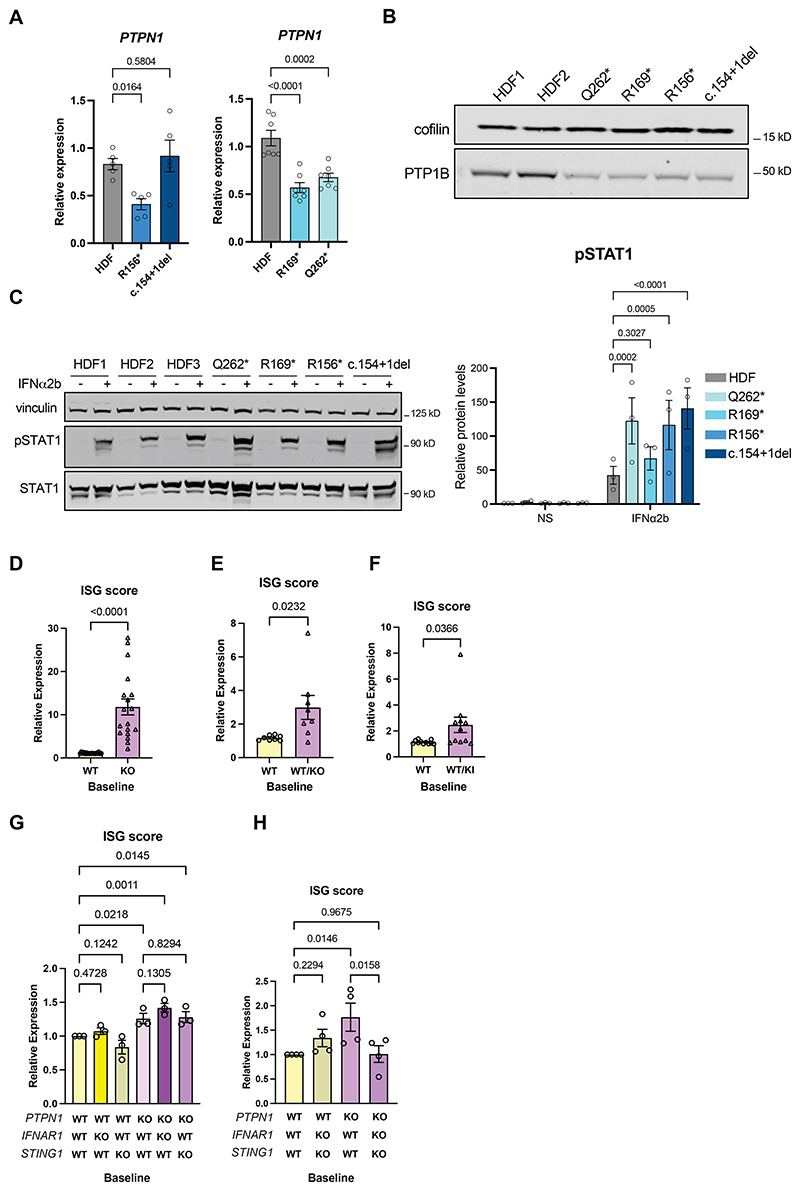
Studies of patient-derived primary fibroblasts and *PTPN1* deficient cells (A) qPCR analysis of *PTPN1* mRNA expression, and (B) representative immunoblots of PTP1B protein expression, in patient-derived primary dermal fibroblasts carrying the indicated heterozygous variants. HDF = control human dermal fibroblasts. For qPCR, n=5-7 experiments and one-way ANOVA with Fisher’s LSD test was used to compare the expression levels of *PTPN1* in healthy control cells and patient cells. (C) Representative immunoblot of phospho-STAT1 (p-STAT1), STAT1 and loading control vinculin in lysates of primary fibroblasts stimulated for 15 min with IFNα2b, and quantification of phospho-STAT1 signal over vinculin signal, averaging control HDF lines. N=3 experiments and mixed model with Dunnett’s multiple comparison test was performed. (D-F) qPCR analysis of the expression of five representative interferon (IFN) stimulated genes (ISGs) (*IFI27, IFI44L, MX1, OAS1* and *RSAD2*), with an ISG score calculated as the median of the expression of these genes in BJ-5ta human fibroblast cell clones wild-type (WT), knock-out (KO) (D), WT/KO (E) or WT/knock-in (KI) for the c.63+1G>C nucleotide substitution (F). Each circle or triangle (D-F) represents the average ISG score from independent clones of the same genotype (WT, two to four clones; WT/KO, four clones; WT/KI, one clone) in one experiment. ISG score is calculated as above. Unpaired t test (D-F) was used to compare the baseline ISG expression levels in WT and mutant BJ-5ta clones. (G) qPCR of baseline ISG expression on a *PTPN1* WT or KO background in hTERT RPE-1 cells, and either WT or single KO of *IFNAR1* or *STING1*. (H) qPCR of baseline ISG expression on a *PTPN1* WT or KO background in hTERT RPE-1 cells, and cells WT or double KO for *IFNAR1* and *STING1*. ISG score is calculated as the median of the expression of four ISGs, *IFI27, IFI16, MX1* and *IRF7*. N=3-4 experiments and one-way ANOVA (G-H) was used to compare the levels of baseline ISG expression in the indicated genotypes.

Consistent with our genetic data, we recorded reduced expression of *PTPN1* mRNA (Figure 4A), and of encoded PTP1B protein (Figure 4B), in the three patient fibroblast cell lines available to us carrying a predicted nonsense mutation in *PTPN1*. Of note, Arg156* was previously shown to be unstable when expressed in HEK293 cells in a study of lymphoma. *PTPN1* mRNA expression was apparently preserved in the patient with the c.154+1del canonical donor splice site variant in intron 2 (Figure 4A). Indeed, deep sequencing of captured *PTPN1* cDNA from primary fibroblasts of this patient revealed skipping of exon 2 in 50% of transcripts (8036 reads versus 7931 reads for the canonically spliced transcript), suggesting preserved expression of the mutant allele and the absence of nonsense-mediated decay (NMD) (Supplementary Figure 2A). However, the skipping of exon 2 leads to a frameshift, and the introduction of a premature stop codon in exon 3 after 19 amino acids. The putative translated peptide would have a size of 40 amino acids and 4.6kDa (Supplementary Figure 2F). Our deep sequencing also revealed a transcript which used an alternative splicing site in exon 2 (2668 reads) (Supplementary figure 2B) with a premature stop codon immediately downstream, leading to a putative peptide of 46 amino acids. We could not detect either truncation by western blotting of primary fibroblast lysates, a finding consistent with protein instability and the observed reduced protein expression (Figure 4B). In contrast, on sequencing of *PTPN1* cDNA captured from whole blood in the context of the c.63+1G>C variant, we observed fewer reads (5122 reads for the c.63+1G>C variant versus 9723 reads in the control sample) and no major splicing defect, indicative of NMD of the mutated allele (Supplementary Figure 2C). We also observed the appearance of a novel minor splicing site in intron 1 (Supplementary figure 2D, 2E), which could lead to retention of part of intron 1, a premature stop codon and the production of a 114 amino acid peptide (Supplementary Figure 2F).

Given evidence of enhanced type I IFN signalling in patient blood, CSF and brain tissue, we tested IFN signalling in patient fibroblasts in vitro. Upon stimulation with IFNα2b, we detected higher levels of phosphorylated STAT1 (p-STAT1) compared to control fibroblasts (Figure 4C). We then investigated the link between a loss of PTP1B function and type I IFN signalling in vitro. Using CRISPR-Cas9 gene editing in BJ-5ta human fibroblasts (an hTERT immortalised cell line commonly employed in studies of innate immune sensing and type I IFN signalling), we generated *PTPN1* homozygous KO single-cell clones (Supplementary Figure 3A). These cells demonstrated significantly enhanced ISG expression at baseline compared to WT control cells, assessed both by targeted qPCR (Figure 4D), and by bulk RNA-Seq (Supplementary Figure 3B). Since we considered haploinsufficiency to be the likely molecular mechanism underlying the disease in our patient cohort, we also generated heterozygous WT/KO BJ-5ta clones, and a clone WT/heterozygous knock-in (WT/KI) for the canonical donor splice site c.63+1G>C variant seen in AGS761 (which leads to a splicing defect and loss of mRNA expression). As expected, and like patient primary fibroblasts, WT/KO and WT/KI cells displayed intermediate levels of PTP1B expression (Supplementary Figure 3C). Similar to complete *PTPN1* KO cells, albeit to a lesser degree, WT/KO (Figure 4E) and WT/KI (Figure 4F) heterozygous clones exhibited elevated baseline ISG expression compared to WT controls, suggesting enhanced IFN signalling.

PTP1B has been implicated in the dephosphorylation of TYK2, a component of the ubiquitously expressed type I IFN receptor (IFNAR) complex, and in the degradation of STING (encoded by *STING1*), an adaptor protein central to cytosolic DNA sensing, both of which could lead to enhanced type I IFN signalling at baseline and upon stimulation (Supplementary Figure 3D). Notably, *PTPN1*-deficient cells demonstrated higher global phospho-tyrosine protein levels even at baseline (Supplementary Figure 3E, 3F, 3G), indicative of a general failure of phosphatase activity. STING levels were also higher in unstimulated *PTPN1* KO cells (Supplementary Figure 4E). Thus, to further explore the negative regulation of type I IFN signalling by PTP1B, we stimulated *PTPN1* WT, KO, WT/KO and WT/KI BJ-5ta cells with either IFNα2b or the STING agonist diABZI. We detected significantly increased IFN signalling after both IFNα2b and diABZI stimulation, as shown by the measurement of global phospho-tyrosine protein levels (Supplementary Figure 3E, 3F, 3H, 3I), ISG expression (Supplementary Figure 4A, 4B) and phosphorylated STAT1 (p-STAT1) (Supplementary Figure 4D). Consistently, *PTPN1* WT/KO and WT/KI clones also demonstrated increased ISG expression following IFNα2b and diABZI stimulation (Supplementary Figure 4A, 4B). Of note, phosphorylated STING (p-STING) protein and *IFNB1* mRNA levels were also induced at a higher level upon diABZI stimulation, suggesting enhanced sensitivity of the STING pathway upstream of IFNAR signalling (Supplementary Figure 4C, 4E).

To distinguish the effects of *PTPN1* deficiency on IFNAR and STING activity, we generated *STING1* KO and *IFNAR1* KO cell pools on a *PTPN1* WT or KO background in hTERT immortalised RPE-1 cells (Supplementary Figure 5A), in which we also observed ISG upregulation in the absence of PTP1B (Supplementary Figure 5B). RPE-1 cells are also commonly used in innate immune sensing studies, and we employed this alternative cell line since we found BJ-5ta fibroblasts were intolerant of multiple rounds of CRISPR gene editing. Western blot confirmed successful KO of *STING1* / *IFNAR1* in *PTPN1* WT and KO cells (Supplementary Figure 5C). Assessing baseline ISG expression in these double-KO cell lines indicated that neither *IFNAR1* nor *STING* KO alone rescued the enhanced ISG expression seen in *PTPN1* deficient cells (Figure 4G). In contrast, with combined deletion of *IFNAR1* and *STING1*, ISG expression levels were equivalent to those seen in *PTPN1* WT cells (Figure 4H), consistent with an effect of PTP1B on both IFNAR1 and STING function.

## Discussion

In this study we identified 12 patients from 11 families harbouring one of ten distinct novel or very rare heterozygous mutations in *PTPN1*, with a variant occurring de novo in three individuals. Importantly, six of these variants introduce premature stop codons, and two involve canonical donor splice site nucleotides which we show to result in abnormal splicing. We provide evidence that these variants are loss of function, noting that *PTPN1* is a highly constrained gene intolerant of null variants (with < 40 out of ~750,000 individuals in total harbouring such variants annotated on gnomAD v4.1.0). Further, both missense variants identified by us have been shown to be important in the allosteric regulation of PTP1B activity.^19,20^ Finally, we observed the same R156* mutation, not present on > 1,600,000 alleles recorded in gnomAD v4.1.0, in two unrelated patients, in one of whom the variant occurred de novo. Given these molecular data, we are confident that haploinsufficiency for PTP1B represents a novel human Mendelian disease.

It is of note that we identified nine asymptomatic mutation-positive parents in our study. The deleterious, or protective, effect of other genetic variants, epigenetic modifications and environmental factors might all influence disease penetrance and expressivity, with clinical non-penetrance an increasingly recognised phenomenon in Mendelian disorders in general,^21^ and immunological diseases in particular.^22^ Notably, clinical non-penetrance has been described in both autosomal dominant and recessive mutant genotypes associated with enhanced type I IFN signalling.^16,23,24^ Further, three such instances of clinical non-penetrance were reported in the context of autosomal dominant *PTPN2* LOF mutations.^13^ At this time, we cannot rule out the possibility that clinically asymptomatic individuals are at risk of disease in later life (or that patients affected during childhood might experience recurrent episodes of loss of skills). Finally, we note that two of the variants that we observed have been described as somatic driver mutations in B cell lymphomas.^5^ We did not record any cancers in the cohort of individuals that we ascertained, and there was no family history of such. However, this observation should be kept in mind during the long-term follow-up of affected individuals and their clinically asymptomatic mutation-positive relatives.

The phenotype of the 12 patients described here is remarkably stereotyped, being characterised by subacute loss of skills following initially normal development, spastic-dystonia, bulbar involvement, preserved head circumference and an absence of seizures. Seven patients were noted to demonstrate an asymmetry of clinical signs at initial presentation, and in four of these individuals there was asymmetry of the cerebral atrophy that was seen on neuroimaging in nine of 12 patients overall. In the context of such asymmetry, the absence of seizures is notable in differentiating this disease from Rasmussen encephalitis, while the lack of neuropsychiatric features, and negative testing for neural antibodies in seven patients, also distinguishes the novel disorder that we describe from antibody-mediated encephalitis.

The observation of enhanced type I IFN signalling in patient blood and CSF, and of increased levels of CSF neopterin,^25,26^ suggests that PTP1B haploinsufficiency can be classified as a novel type I interferonopathy.^27^ Indeed, the paradigm type I interferonopathy Aicardi-Goutières syndrome (AGS) was considered as a possible diagnosis in a number of the cases described here. While AGS classically presents in the first year of life with white matter involvement, intracranial calcification and cerebral atrophy, later onset cases with normal neuroimaging or nonspecific white matter changes, with or without intracranial calcification, are well described. Features apparently distinguishing PTP1B-related encephalopathy from AGS are a later age at onset (nine of 12 cases in our cohort presenting beyond 18 months of age), notable bulbar involvement manifesting as difficulties with swallowing and expressive speech, and cerebral atrophy as the predominant neuroradiological sign.

Considering the likely inflammatory basis of AGS, a few reports have described the use of ‘broad spectrum’ immunosuppression in affected individuals,^28,29^ without obvious clinical efficacy. In contrast, in six of eight treated cases described here, it was judged clinically that neurological status improved in association with high dose corticosteroids (+/- intravenous immunoglobulin, azathioprine and mycophenolate mofetil) (including three patients previously reported by Sa et al.,^14^ all of whom were found to harbour mutations in *PTPN1* in this study). However, it is also the case that all 12 patients demonstrated clinical stabilisation, even if most were left with major neurological deficits, and there was complete, or near complete, resolution of the cerebral atrophy and/or white matter disease seen in the initial stages of the disease, irrespective of treatment status. Thus, the precise relationship of steroid therapy to clinical outcome in PTP1B haploinsufficiency remains uncertain. Again relating to treatment, a possible clinical implication of our work would be to use JAK1 / TYK2 inhibition as a therapeutic strategy (with JAK1 and TYK2 components of the type I IFN receptor complex). To our knowledge, no patients with haploinsufficiency of PTP1B have yet been treated in this way. One possible difficulty with this approach would be the known limited penetration of such drugs into the central nervous system.

In a high-throughput screening assay involving gene silencing in mouse embryonic fibroblasts, with follow-up experiments in human monocyte-derived dendritic cells, PTP1B was identified as a negative regulator of type I IFN signalling after DNA stimulation.^6^ A number of possible mechanisms have been suggested to explain this negative regulatory function of PTP1B, including through roles in TYK2 dephosphorylation and STING proteasomal degradation.^7,8^ Our patient and in vitro data emphasise the importance of PTP1B in the control of STING and JAK-STAT signalling in human immune homeostasis.^3^ By extrapolation, given that PTP1B inhibitors are in active development for a variety of indications,^2^ our findings indicate a need for careful neurological monitoring with the use of such inhibition.

The present study is limited by the relatively small number of patients ascertained and the retrospective nature of the analysis. The identification of more patients will provide important information on the breadth of phenotype associated with PTP1B haploinsufficiency, and a better understanding of the appropriate management and treatment of this disorder. Prospective follow-up will also provide new knowledge on the risk, if any, of further episodes of neurological loss of skills and other phenotypes (particularly myeloma) in symptomatic patients, and of individuals asymptomatic into adulthood manifesting later onset disease.

## Supplementary Material

Supplementary

## Figures and Tables

**Table 1:** Summary of molecular data by family

Family	cDNA variant	Consequence	Inheritance	gnomAD v4.1.0
**AGS492**	c.590A>G (exon 6)	p.(Lys197Arg)(K197R)	Paternally inherited (x2)*	0
**AGS761**	c.63+1G>C (intron 1)	Partial intron 1 retention and NMD	De novo	0
**AGS1036**	c.466C>T (exon 5)	p.(Arg156*)(R156*)	Maternally inherited	0
**AGS1312**	c.1000delG (exon 8)	p.(Val334*)(V334*)	Paternally inherited	0
**AGS1421**	c.370_386del (exon 5)	p.(Tyr124Argfs*7)(Y124Rfs*7)	Paternally inherited	0
**AGS2942**	c.466C>T (exon 5)	p.(Arg156*)(R156*)	De novo	0
**AGS3148**	c.154+1del (intron 2)	Exon 2 skipping	De novo	0
**AGS3165**	c.784C>T (exon 7)	p.(Gln262*)(Q262*)	Maternally inherited	0
**AGS3479**	c.166C>T (exon 3)	p.(Arg56Trp)(R56W)	Maternally inherited	2/1,612,624 alleles
**AGS3542**	c.505C>T (exon 6)	p.(Arg169*)(R169*)	Paternally inherited	1/1,613,702 alleles
**AGS3561**	c.619G>T (exon 6)	p.(Glu207*)(E207*)	Paternally inherited	0

NMD = nonsense-mediated decay

* Two first cousins related through their unaffected fathers

p.(K197R) and p.(Arg56Trp) reported to be involved in the allosteric regulation of PTP1B function.^19,20^ p.(Arg56Trp) and p.(Arg156*) reported as somatic mutations in, respectively, Hodgkin lymphoma and primary mediastinal B cell lymphoma.^5^

**Table 2:** Age at onset, clinical features, neuroradiology, treatment effect and status at last contact by individual

Patient (sex)	Age at onset	Clinical features	Neuroradiology (age in months)	Treatment effect	Status (GMFCS;^30^ CFCS^31^ at last contact)
**AGS492.1 (M)**	2.5y	Rapid onset of L-sided hemiparesis evolving to SD tetraparesis over several months becoming unable to sit without support and poor head control. No information on HC	Bilateral WMD (31); BG calcification (31); Repeat MRIs showed slight progression of WMD (40; 44); no further follow-up imaging available	No clear improvement with oral prednisolone 1mg/kg/day at age 3y 9m for 3m	Condition stabilised; alive age 11y (GMFCS V; CFCS IV)
**AGS492.4 (M)**	3.5y	Rapid onset of R-sided weakness evolving to bilateral spasticity, loss of walking, dysphasia, and dysphagia over several weeks. HC 25^th^ centile	WMD L>R with BG calcification (44); more profuse WMD with CA (46); marked improvement in WM signal (96)	MP (30mg/kg for 3d repeated monthly for 3m) started in first few weeks after presentation associated with resumption of limited ambulation and less drooling	Condition stabilised; alive age 8y (GMFCS III; CFCS IV)
**AGS761 (M)**	15m	Fever with irritability and rapid loss of motor skills, language and swallowing (requiring gastrostomy) over days, becoming SD with truncal hypotonia. Raised LFTs at presentation. HC 9^th^ centile	Patchy WMD and CA (16)	IVIG for 6m with no obvious effect	Condition stabilised; alive age 8y (GMFCS V; CFCS IV)
**AGS1036** **(F) (Case 2 in Sa et** **al.)^14^**	5y	Subacute onset over 3m of L- sided weakness, frequent falls and difficulties climbing stairs, also becoming dysarthric with dysphagia and drooling. Subsequent SD LL>UL, L>R. Normal HC. Raised LFTs at presentation	CA (R>L) (5m after symptom onset); MRI 23m after Rx initiation (35m after symptom onset) showed significant reversal of CA	IV MP (4 courses 30mg/kg/d for 5 days and IVIG 2g/kg 3 – 4m apart) started 12m after symptom onset associated with significant improvement in all domains so that she was left with only residual LL hypertonia	Condition stabilised; alive age 13y (GMFCS I; CFCS I)
**AGS1312** **(F) (Case 3 in Sa et** **al.)^14^**	4y	Mild motor delay (walked 21m) then progressive loss of walking leading to use of wheelchair outside and complete loss of expressive language over 1y with dysphagia. SD R>L. HC <0.4^th^ centile (no prior measures). Marked improvement in LL tone associated with Rx so that she regained independent ambulation albeit with a spastic paraplegia, and speech and swallowing returned to normal. Raised LFTs at presentation	Mild global CA (9m after symptom onset); MRI 13m after Rx initiation (and 30m after symptom onset) showed complete normalisation	Improved with IV MP (3 days, 30mg/kg/d and IVIG 2g/kg for 5 courses over 20m started 15m after symptom onset	Condition stabilised; alive age 12y (GMFCS II; CFCS I)
**AGS1421** **(M) (Case 1 in Sa et** **al.)^14^**	22m	Progressive L-sided hemiparesis extending bilaterally with SD (LL>UL) over months, associated with fever, loss of speech and dysphagia. HC normal. Raised LFTs at presentation	R CHA (~27) extending bilaterally (34)(12m after symptom onset); MRI normal (76)(26m after treatment)	Improved fine motor and non-verbal skills with 6 weekly IV MP 30mg/kg/d and IVIG 2g/kg started 18m after symptom onset	Condition stabilised; alive age 11y (GMFCS IV; CFCS III)
**AGS2942 (F)**	8y	Appearance of L LL spasticity when running, followed by cognitive decline, dysarthria and recurrent fevers over 1y. Subsequently regained all skills. Normal HC	Modest WMD and CA (114); normalisation of CA and reduction of WMD (125)	Not treated	Condition stabilised and almost returned to pre- morbid state; alive age 10y (GMFCS I; CFCS I)
**AGS3148 (M)**	15m	Febrile seizure followed 1m later by loss of walking and speaking abilities so that by age 19m speech was completely absent he could not walk, was tetra- spastic and demonstrated bulbar involvement (dysphagia). HC 25- 50^th^ centile	WM T2 high signal (16); reduction (33) and almost normalisation of WMD (168)	Not treated	Condition stabilised; alive age 20y (GMFCS V; CFCS IV)
**AGS3165 (M)**	4.5y	Febrile episode followed by irritability, spasticity, dysarthria and dysphagia progressing rapidly so that within 6m he was unable to walk, speech was incomprehensible and unsafe swallow. Normal HC	Normal MRI (54m); WMD R>L (58); WMD more diffuse (58); almost complete resolution of WMD (61)	Bolus corticosteroids (1mg/kg/day) age 58m without major clinical benefit; further corticosteroid (1mg/kg/day) aged 63m associated with a definite improvement in walking, albeit with SP, speech and swallowing	Condition stabilised; alive age 7y (GMFCS II; CFCS II)
**AGS3479 (F)**	18m	L-sided hemiparesis progressing to 4-limb SD with dysphagia and complete loss of speech. HC 25^th^ centile (75^th^ centile 12m earlier)	Initial R CHA (19); atrophy becoming bilateral in the absence of any WMD (22); no further imaging undertaken	IVIG stopped almost immediately due to an allergic reaction. No further treatment initiated	Condition stabilised; alive age 3y (GMFCS V; CFCS V)
**AGS3542 (F)**	23m	Progressive spastic tetraparesis (L>R) with preserved cognition and speech, experiencing a further period of regression at age 36m also affecting speech and swallowing. Now has <20 words but communicates well with a computer and can use an electric wheelchair unassisted. Normal HC	Initial R CHA with subtle WMD L>R (26); extending bilaterally (44); almost normalised brain volume (60; 92)	Definite response to pulsed MP 20mg/kg/d for 5d monthly for 3m starting 27m, and to a further dose (20mg/kg/d for 3d weekly for 4w at ages 38m, 51m and beyond because of clinical deterioration	Condition stabilised; alive age 8y (GMFCS IV; CFCS III)
**AGS3561 (F)**	7y	Appearance over several weeks of gait and speech disturbance with mild cognitive decline, evolving to SD R>L with dysarthria and dysphagia. Subsequently judged to show re- attainment of some skills so that now able to speak in simple sentences and can walk independently over short distances. Normal HC	WMD with CA (92); CA becoming more prominent (96); no further follow-up imaging available	Not treated	Condition stabilised with some re-attainment of skills; alive age 10y (GMFCS II; CFCS II)

BG: basal ganglia; CA: cerebral atrophy; CHA: cerebral hemiatrophy; CSF: cerebrospinal fluid; CFCS: communication function classification system; d: day; F: female; GMFCS: gross motor function classification system; HC: head circumference; IVIG: intravenous immunoglobulin; L: left; LFTs: liver function tests; LL: lower limbs; M: male; m: month; MP: methylprednisolone; NA: not assessed; R: right; Rx: treatment; SD: spastic dystonia; SP: spastic paraparesis; UL: upper limbs; w = weekly; WM: white matter; WMD: white matter disease; y: year

## Data Availability

Exome sequencing data are not publicly available due to the possibility of compromising privacy. Human fibroblasts are primary cells and therefore a limited resource. Availability is through the corresponding authors subject to technical constraints and completion of a material transfer agreement required to ensure patient privacy.

## Research in context

### Evidence before this study

We searched PUBMED, bioRxiv and MedRxiv for articles published in English from database inception to August 1, 2024, using the terms “PTPN1” and “mutation”. This search showed that while somatic mutations in PTP1B have been described in Hodgkin lymphoma and primary mediastinal B cell lymphoma, germline mutations have not been previously reported. We also searched the same databases using the terms “PTPN1” and “interferon”, and identified reports describing variable roles for PTP1B in the regulation of type I interferon (IFN) signalling in cell models.

### Added value of this study

Our study describes, to our knowledge for the first time, the phenotypic consequences of PTP1B haploinsufficiency in humans. Specifically, heterozygous loss-of-function mutations in *PTPN1* are associated with a highly stereotyped neuroradiological phenotype associated with enhanced type I IFN signalling. Our ex vivo and in vitro experiments show that partial loss of PTP1B function is sufficient to impair negative regulation of type I IFN signalling.

### Implications of all the available evidence

PTP1B, encoded by *PTPN1*, is a ubiquitously expressed protein tyrosine phosphatase involved in the regulation of a broad range of signalling pathways. Our study reveals haploinsufficiency for PTP1B as a novel cause of disease in humans. The observed *PTPN1* variants lead to reduced levels of protein and a disturbance of type I IFN signalling negative regulation. Notably, the associated autoinflammatory encephalopathy presents as subacute loss of skills, which may be responsive to immune suppression in some cases.
