# Supplementary material for: Autoinflammatory encephalopathy due to PTP1B haploinsufficiency: a case series
Source: Lancet Neurol. Author manuscript; Available in PMC 2025 Mar 1. (PMC7617446; doi:10.1016/S1474-4422(24)00526-X)
Supplement: Supplementary [file EMS203526-supplement-Supplementary.docx]

**SUPPLEMENTARY MATERIAL**

**Autoinflammatory encephalopathy due to PTP1B haploinsufficiency: a case series**

**TABLE OF CONTENTS**

**SUPPLEMENTARY METHODS**

**Samples obtained from patients**

**Genetic studies**

**Protein structural analysis**

**IFN status**

**Cell culture and reagents**

**CRISPR-Cas9 gene editing**

**RNA extraction, reverse transcription and qPCR for primary cells and cell lines**

**Western blotting**

**Bulk RNA sequencing of BJ-5ta clones and analysis**

**Statistics**

**CLINICAL HISTORIES**

**SUPPLEMENTARY FIGURES AND TABLES**

**SUPPLEMENTARY TABLES**

**SUPPLEMENTARY REFERENCES**

**SUPPLEMENTARY METHODS**

**Study design**

Patients were ascertained clinically via paediatric neurologists and clinical geneticists internationally, both through direct clinical contacts and the use of Genematcher.^1^ Research testing was undertaken in the MRC Human Genetics Unit, Institute of Genetics and Cancer, University of Edinburgh, and in the Imagine Institute, Paris. Sex was defined clinically by the physician responsible for the care of the patient. In the absence of any suggestion of the existence of population founder mutations in our cohort, data on race/ethnicity are not included in this study.

**Samples obtained from patients**

Samples for research testing in the MRC Human Genetics Unit, Institute of Genetics and Cancer, University of Edinburgh, and in the Imagine Institute, Paris were obtained from the probands and parents with written informed consent; respectively covered by approvals from the Leeds (East) Research Ethics Committee (10/H1307/132) and the Comité de Protection des Personnes (ID-RCB/EUDRACT: 2014-A01017-40).

Clinical status at last contact was scored according to the Communication function classification system (CFCS) and Gross motor function classification system (GMFCS).^2,3^

**Genetic studies**

DNA was extracted from whole blood using standard methods. Exome sequencing was performed on genomic DNA using a SureSelect Human All Exon kit (Agilent Technologies) for targeted enrichment, and Illumina HiSeq2000 for sequencing. Variants were assessed using the in silico programs SIFT (http://sift.jcvi.org) and Polyphen2 (<http://genetics.bwh.harvard.edu/pph2/>), and the splicing module of Alamut® Visual. Population allele frequencies were obtained from the gnomAD v4.1.0 database (http://gnomad.broadinstitute.org). Sanger sequencing was performed to confirm the identified *PTPN1* variants. The reference sequence used for primer design and nucleotide numbering was ENST00000371621.5 / NM_002827.4 / NP_002818.1.

**Protein structural analysis**

The highest resolution crystal structure of the apo form was used for visualisation and modelling.^4^ The A chain was used for visualisation with ChimeraX .^5^ The effects of missense variants on protein stability were modelled using FoldX.^6^ By taking the average over three separate replicates of both chains present in the asymmetric unit, the ΔΔG was calculated to be 1.1 and 0.7 kcal/mol for R56W and K197R, respectively, suggesting that neither variant is likely to be highly destabilising.

**IFN status**

Whole blood was collected into PAXgene tubes (Qiagen) and total RNA extracted using a PreAnalytix RNA isolation kit. IFN scores were generated in one of two ways as previously described.^7,8^ Using TaqMan probes to measure the mRNA expression by qPCR of six ISGs (*IFI27, IFI44L, IFIT1, ISG15, RSAD2,* and *SIGLEC1*) normalized to the expression level of *HPRT1* and *18S rRNA*: here, the median fold change of the ISGs constitutes an IFN score for each individual, which is compared with the IFN score of 29 healthy controls, with an abnormal score being defined as greater than the mean of controls +2 SD. Scores above this value (2.466) are designated as positive. For NanoString ISG analysis, total RNA was similarly extracted from whole blood with a PAXgene (PreAnalytix) RNA isolation kit. Analysis of 24 genes of interest (*IFI27, IFI44L, IFIT1, ISG15, RSAD2, SIGLEC1, CMPK2, DDX60, EPSTI1, FBXO39, HERC5, HES4, IFI44, IFI6, IFIH1, IRF7, LAMP3, LY6E, MX1, NRIR, OAS1, OASL, OTOF,* and *SPATS2L*) and 3 housekeeping genes (*NRDC, OTUD5,* and *TUBB*) was conducted using the NanoString customer designed CodeSets according to the manufacturer’s recommendations (NanoString Technologies). Agilent Tapestation was used to assess the quality of the RNA. 100 ng total RNA was loaded for each sample. Data were processed with nSolver software (NanoString Technologies). The data were normalised relative to the internal positive and negative calibrators, the three reference probes, and the control oligo sample. The median of the levels for the 24 probes for each of 41 healthy control samples and for each patient sample was calculated to determine the NanoString IFN score. The mean NanoString IFN score of the 41 healthy controls +2 SD of the mean was calculated, and scores above this value (2.758) were designated as positive.

**Cell culture and reagents**

Primary human dermal fibroblasts derived from healthy controls (HDF) and patients, and BJ-5ta hTERT immortalised fibroblasts (CRL-4001, ATCC, RRID: CVCL_6573) were cultured in Dulbecco’s modified Eagle’s medium (DMEM), high glucose, GlutaMAX supplement, pyruvate (31966047, Gibco) supplemented by 10% fetal calf serum (FCS) and 1% penicillin/streptomycin (p/s). hTERT RPE-1 cells were a generous gift from Prof. Andrew P Jackson Laboratory, MRC HGU, the University of Edinburgh, and were originally purchased from ATCC (CRL-4000, RRID: CVCL_4388). hTERT RPE-1 cells were cultured in Advanced DMEM/F-12 (12634010, Gibco) supplemented by 10% FCS, 1% p/s and 1X GlutaMAX (35050038, Gibco). BJ-5ta cells were maintained in a 37°C, 5% CO_2_ and 3% O_2_ hypoxic incubator while primary human dermal fibroblasts and hTERT RPE-1 cells were maintained in a 37°C, 5% CO_2_ incubator. Cells were routinely checked for the absence of mycoplasma contamination with Mycoplasma PCR Detection Kit (ab289834, abcam). Dose and duration for stimulation of cells with IFNα2b (11105, PBL Assay Science) and diABZI (28054, Cayman Chemical) were specified for each experiment in the figure legends.

**CRISPR-Cas9 gene editing**

Direct delivery of ribonucleoprotein (RNP) complex consisting of Cas9 protein and single guide RNA (sgRNA) was achieved by electroporation. For gene knock-out (KO), CRISPR Gene Knockout Kit v2 from Lonza comprising three guides targeting one gene is used. sgRNA sequences for *PTPN1* KO were UAAGAUCUCUCGAGUUUCUU, AAGAAUGAGGCUGGUGAUUC and CCCGGAGCACGGGCCCGUUG; sgRNA sequences for *STING1* KO were CGGGAUGGAUGGAUGCAGGC, UUAACAGCAGUCCCAGCUGC and CCUGCCUGGUGACCCUUUGG; sgRNA sequences for *IFNAR1* KO were UUUACUUUAAAGAACUGGGA, GAGUGAAGAAAAGUUGCAUU and AAACACUUCUUCAUGGUAUG. sgRNA(s) and Alt-R S.p. Cas9 Nuclease 3NLS (1081059, IDT) were gently mixed and incubated in room temperature for 15 minutes for RNP complex formation, which later was introduced into the cells via P2 Primary Cell 4D-Nucleofector™ X Kit L (V4XP-2024, Lonza) for BJ-5ta or P3 Primary Cell 4D-Nucleofector® X Kit L (V4XP-3024, Lonza) for hTERT RPE-1. Electroporation was performed with 4D-Nucleofector® X Unit (AAF-1003X, Lonza). Programs for BJ-5ta and hTERT RPE-1 were CZ-167 and EA-104, respectively. For *PTPN1* knock-in (KI) of variant c.63+1G>C by homologous recombination, an sgRNA (sequence GCGCTCCCGCACCTGGTAAA) and a single-stranded oligodeoxynucleotide (ssODN, sequence GGCCGAGAAGGAGGCGCAGCAGCCGCCCTGGCCCGTCATGGAGATGGAAAAGGAGTTCGAGCAGATCGACAAGTCCGGGAGCTGGGCGGCAATTTACCAGcTGCGGGAGCGCCCCGGAGCGTGGCGGGCCCTTCGCTTAGGCCGCTTGAACATCCCCTCAGACCTCCAGGCCCCAGACTCCCTCTGGGTCTTGCCCTCTG) serving as a repair template were used.

To generate BJ-5ta clones, two days post electroporation, CRISPR-ed pools were single-cell sorted into a 96-well plate via flow cytometry. After cells reached confluency, a PCR-based selection method was used to identify clones with the desired genotypes. Primer sequences for *PTPN1* were GAAGCAGCAGCGGCTAGG (forward) and CCTCAAGCAGTAGGAGCGAG (reverse). Successful gene-editing for either pools or single-cell clones was first assessed by the Synthego ICE CRISPR Analysis Tool, then followed by western blot confirmation.

**RNA extraction, reverse transcription and qPCR for primary cells and cell lines**

RNA from primary fibroblasts or cell lines was extracted using RNAqueous-Micro Kit (for primary fibroblasts, AM1931, Ambion) RNeasy Micro Kit (for other cell lines, 74004, Qiagen) following the manufacturer’s instructions. Reverse transcription of extracted RNA was performed using High-Capacity cDNA Reverse Transcription Kit (4368814, Thermo Fisher Scientific). The expression of genes of interest, relative to the geomean of the expression of housekeeping genes (*ACTB* and *HPRT1* for BJ-5ta and *ACTB*, *GAPDH* and *HPRT1* for hTERT RPE-1), was analysed by TaqMan quantitative real-time PCR (TaqMan Fast Universal PCR Master Mix (2 ×) No AmpErase UNG, 4352042, Thermo Fisher Scientific) on a QuantStudio 5 real-time PCR machine (A28140, Applied Biosystems). TaqMan probes used were *PTPN1* (Hs00942477_m1), *IFNB1* (Hs01077958_s1), *IFI16* (Hs00986757_m1), *IFI27* (Hs01086370_m1), *IFI44L* (Hs00199115_m1), *MX1* (Hs00895608_m1), *OAS1* (Hs00973637_m1), *RSAD2* (Hs01057264_m1), *IRF7* (Hs00185375_m1), *ACTB* (Hs01060665_g1), *GAPDH* (Hs02786624_g1) and *HPRT1* (Hs03929096_g1). The ∆∆Ct method was adopted to calculate the relative levels of gene transcription, relative to the mean values of the healthy controls or wild-type controls. The calculation method for an ISG score is specified in the figure legends.

**Western blotting**

BJ-5ta and hTERT RPE-1 cells were incubated for 1 h at 4°C with rotation in lysis buffer (25 mM Tris–HCl, pH 8.0, 1% NP-40, 150 mM NaCl, 1.5 mM MgCl_2_, 0.05% SDS, 0.5% sodium deoxycholate, supplemented with 750 U/mL benzonase nuclease (E1014-25KU, Scientific Laboratory Supplies), protease inhibitor cocktail (04693159001, Roche) and phosphatase inhibitor cocktail (04906837001, Roche)) to obtain whole-cell lysates (WCLs). WCLs were then subjected to centrifugation at 12,000 rpm at 4°C for 10 min to remove insoluble fraction. Supernatant containing soluble protein fraction was collected, and total protein concentration was measured using Pierce BCA protein assay kit (23227, Thermo Fisher Scientific). Same amount of protein from each sample with Pierce Lane marker reducing sample buffer (39000, Thermo Fisher Scientific) was denatured at 95°C for 10 min and resolved on NuPage 4–12% Bis–Tris Gels (NP0336BOX, Invitrogen) in 1X NuPage MOPS SDS running buffer (NP0001, Invitrogen). Proteins were then transferred from the gel onto the nitrocellulose membrane of an iBlot 2 NC Regular Stack (IB23001, Invitrogen) for 15 min at 15 V using the iBlot 2 Dry Blotting System (IB21001, Invitrogen). Proteins were extracted from primary fibroblasts using radioimmunoprecipitation assay lysis buffer (#89900; Life Technologies) supplemented with 1 % protease inhibitor (Halt Protease Inhibitor Cocktail, Life Technologies) and 1 % phosphatase inhibitor (Phosphatase Inhibitor Cocktail 2, Sigma-Aldrich). Bolt LDS Sample Buffer (Novex; Life Technologies) and Bolt Sample Reducing agent (Novex; Life Technologies) were added to protein lysates, denatured at 70°C for 10 min. Protein extracts were then resolved on 4–12 % Bolt Bis-Tris Plus gels (Invitrogen) and transferred to nitrocellulose membranes (iBlot Invitrogen). Membranes were blocked in intercept (TBS) blocking buffer (927–60001, LI-COR) for 30 min at room temperature and incubated overnight at 4°C with rotation with primary antibodies of interest diluted in blocking solution supplemented with 0.1% Tween 20 (EC-607, National Diagnostics). Primary antibodies used in this study were Vinculin (13901S, RRID: AB_2728768), Cofilin (5175S, RRID: AB_10622000), phospho-tyrosine (8954S, RRID: AB_2687925), p-STAT1 (7649S, RRID: AB_10950970), STAT1 (9176S, RRID: AB_2240087), p-STING (19781S, RRID: AB_2737062), STING (13647S, RRID: AB_2732796), all of which are from Cell Signalling Technology; PTP1B (for BJ-5ta and hTERT RPE-1: MABS197, Sigma-Aldrich, RRID: AB_11204000; for primary fibroblasts: ab244207, Abcam, RRID:AB 2877148), STING (66680-1-Ig, Proteintech, RRID: AB_2882034) and IFNAR1 (ab124764, Abcam, RRID: AB_10972855). After washing away unspecific binding of primary antibodies, IRdye-conjugated anti-mouse (926–68070, LI-COR, RRID: AB_10956588) or anti-rabbit (925–32211, LI-COR, RRID: AB_2651127) secondary antibodies diluted in intercept (TBS) blocking buffer were used to detect targeted proteins. Membranes were then scanned using the Odyssey CLx System (LI-COR). Densitometry quantification and analyses were performed using either the Image Studio Lite software v.5.2 (LI-COR) or ImageJ.

Samples to be blotted for IFNAR1 were treated with PNGase F (P0704S, NEB) to remove glycosylation prior to western blotting following manufacturer’s instructions. Briefly, WCLs were obtained using the same lysis buffer as above without 0.05% SDS and 0.5% sodium deoxycholate. Then, samples containing glycoprotein, together with 1X Glycoprotein Denaturing Buffer was denatured at 100°C for 10 min. After chilling on ice for 10 s, samples were mixed with 1X GlycoBuffer 2, 1% NP-40, 25 U/µL PNGase F and incubated at 37°C for 1 h.

**Bulk RNA sequencing of BJ-5ta clones and analysis**

Two independent clones per genotype (WT and KO) and two experimental repeats for each clone were used. Libraries were prepared using the NEBNEXT Ultra II Directional RNA Library Prep kit (7760, NEB), and the Poly-A mRNA magnetic isolation module (E7490, NEB) was used for mRNA enrichment per the manufacturer’s protocol. After library preparation, Sequencing (2x60) was performed on the NextSeq 2000 platform (20038897, Illumina Inc) using NextSeq 2000 P3 Reagents (100 Cycles) (20040559, Illumina Inc). Coverage was relatively even, and the majority of libraries generated ≥45M reads (Min: 41.2M, Max: 57.1M, Mean: 49.7M). RNA sequencing was analysed via nf-core/rnaseq pipeline (v3.14.0) and the obtained length-scaled gene counts were subjected to DESeq2 analysis in R. For gene set enrichment analysis (GSEA), differentially expressed genes (DEGs) were uploaded to GSEA software^9^ and hallmark gene sets was chosen to identify the enriched pathways.

***PTPN1* capture RNA sequencing**

Total RNA was extracted from primary fibroblasts (HDF, AGS3148 and AGS2942) or from whole blood sampled on a Paxgene RNA tube (Ctrl and AGS761) as described above. RNA was quantified using a Qubit Fluorometer and High Sensitivity RNA assay (Thermo Fisher Scientific). Quantities > 800 ng of RNA were used as starting material for the first strand synthesis using the VILO SuperScript III (11754050, Thermo Fischer Scientific). The second strand was synthesised using the Second Strand cDNA Synthesis Kit (A48571, Thermofischer Scientific). Then, 50 ng of cDNA for each sample was prepared with Twist’s unique dual indexes (Twist Bioscience) by steps of fragmentation, end repair, and dA-tailing. Adapters were ligated and indexes were added to the amplified cDNA. Samples were pooled to a total mass of 1500 ng and the hybridisation reaction was performed for 16 h using home-made biotinylated single strand DNA probes designed to cover *PTPN1* gene region. During the capture process, barcoded libraries molecules bound to the biotinylated beads are retained by streptavidin coated magnetic beads on a magnet and amplified by 12 PCR cycles. The purified libraries were then controlled by fluorimetry Qubit Double Strand DNA (Thermo Fischer Scientific) and capillary electrophoresis double strand DNA on the Fragment Analyzer (Agilent Technologies). An equimolar pool of all the libraries was prepared according to these 2 measurements. The concentration of this pool was measured by Q-PCR (KAPA Library Quantification kit, Roche). The libraries were sequenced on the NovaSeq6000, Illumina (S1 FlowCells, PE100, ~30 millions of reads/clusters per library were targeted).

After demultipexing, the reads were aligned to the reference genome GRCh37/Hg19 using the STAR software (version 2.7.5). Following alignment, PCR duplicates were identified and removed using Picard Tools (<https://broadinstitute.github.io/picard/>). Splice junctions on regions of interest were then visualized using the Integrative Genomics Viewer (IGV V2.14.1).

**Statistics**

The statistical testing for each experiment is specified in the figure legends. In Figure 4A, one-way ANOVA was used to compare *PTPN1* mRNA expression levels in healthy donor fibroblasts and patient fibroblasts. n = 5-7. In Figure 4C, mixed-model two-way ANOVA was used to compare the levels of phospho-STAT1 in healthy donor fibroblasts and patient fibroblasts following stimulation with IFNα2b. n = 3. In Figure 4D-F, unpaired *t* test was used to compare the baseline ISG expression levels in WT and mutant BJ-5ta clones. n = 8-18. In Figure 4H-I, one-way ANOVA was used to compare the baseline ISG scores in the distinct genotypes. Specifically in Figure 4H, pair-wise comparisons were assessed in *PTPN1*^WT^/*IFNAR1*^WT^/*STING1*^WT^ and *PTPN1*^WT^/*IFNAR1*^KO^/*STING1*^WT^, *PTPN1*^WT^/*IFNAR1*^WT^/*STING1*^WT^ and *PTPN1*^WT^/*IFNAR1*^WT^/*STING1*^KO^, *PTPN1*^WT^/*IFNAR1*^WT^/*STING1*^WT^ and *PTPN1*^KO^/*IFNAR1*^WT^/*STING1*^WT^, *PTPN1*^WT^/*IFNAR1*^WT^/*STING1*^WT^ and *PTPN1*^KO^/*IFNAR1*^KO^/*STING1*^WT^, *PTPN1*^WT^/*IFNAR1*^WT^/*STING1*^WT^ and *PTPN1*^KO^/*IFNAR1*^WT^/*STING1*^KO^, *PTPN1*^KO^/*IFNAR1*^WT^/*STING1*^WT^ and *PTPN1*^KO^/*IFNAR1*^KO^/*STING1*^WT^, *PTPN1*^KO^/*IFNAR1*^WT^/*STING1*^WT^ and *PTPN1*^KO^/*IFNAR1*^WT^/*STING1*^KO^; in Figure 4I, pair-wise comparisons were assessed in *PTPN1*^WT^/*IFNAR1*^WT^/*STING1*^WT^ and *PTPN1*^WT^/*IFNAR1*^KO^/*STING1*^KO^, *PTPN1*^WT^/*IFNAR1*^WT^/*STING1*^WT^ and *PTPN1*^KO^/*IFNAR1*^WT^/*STING1*^WT^, *PTPN1*^WT^/*IFNAR1*^WT^/*STING1*^WT^ and *PTPN1*^KO^/*IFNAR1*^KO^/*STING1*^KO^, *PTPN1*^KO^/*IFNAR1*^WT^/*STING1*^WT^ and *PTPN1*^KO^/*IFNAR1*^KO^/*STING1*^KO^. In Supplementary Figure 4A-C, ratio paired *t* test was used to compare the ISG scores or *IFNB1* expression levels in WT and mutant BJ-5ta clones following stimulation with IFNα2b or the STING agonist diABZI. n = 3-7. In Supplementary Figure 4D-E, unpaired *t* test was used to compare the levels of phospho-STAT1, phospho-STING, and total STING in WT and KO BJ-5ta clones. n = 4-5. In Supplementary Figure 5B, unpaired *t* test was used to compare the baseline ISG scores in WT and KO RPE-1 cells. n = 14.

**CLINICAL HISTORIES**

**AGS492.1**. Cousin to AGS492.4, related through unaffected fathers, brothers to one another. Normal head circumference (25% centile). Onset at age 2.5 years. Rapid onset of left-sided hemiparesis, with bilateral high T2 white matter changes on cerebral MRI (aged 31 months) and calcification of the basal ganglia mainly on the right (aged 31 months). His clinical phenotype evolved to a spastic-dystonic tetraparesis over several months, becoming unable to sit without support, with poor head control, and no language although his social interaction was relatively maintained. A trial of oral prednisone (1 mg/kg/day, gradually tapered) at age 3 years 9 months for 3 months was associated with no apparent change in his clinical condition. Repeat MRIs at age 40 and 44 months showed a slight progression of the white matter abnormalities (bilateral T2/FLAIR hyperintensity). He was alive at last contact aged 11 years, after which he was lost follow-up.

His father was clinically asymptomatic into adulthood (no other details available).

At 32 months of age CSF neopterin was recorded at 90 nmol/L (normal range 9 – 55 nmol/L), and interferon alpha activity in the CSF and serum was elevated at 6 IU/mL and 9 IU/mL (normal < 3 IU/mL). respectively. ISGs tested on two occasions were also elevated, with interferon scores of 8.131 and 8.009 at ages 3.62 and 4.1 years respectively.

Sequencing identified a paternally inherited heterozygous c.590A>G (p.(Lys197Arg)) (K197R) variant in *PTPN1* (the same variant seen in his affected cousin AGS492.4), which was not present on gnomAD v4.1.0.

**AGS492.4**. Cousin to AGS492.1, related through unaffected fathers, brothers to one another. Aged 3.5 years, he experienced the rapid onset of initially right-sided weakness and spasticity, dysphasia and dribbling. His condition worsened over several weeks so that lost the ability to walk. MRI brain at age 44 months showed bilateral high signal T2 changes in the matter, more prominent on the left. Repeat MRI brain at age 46 months showed progression of the white matter changes which were now more diffuse with definite cerebral atrophy. His last cerebral MRI at age 96 months showed an improvement in the high signal T2 changes. Brain biopsy at this age showed non-specific perivascular inflammatory changes in the cortical white matter and meninges. He was treated with methylprednisolone (30mg/kg for three days repeated monthly for three months) and azathioprine (600 mg/m^2^ bd for 9 months), which was associated with a partial response so that he began to regain some degree of limited ambulation and a reduction of drooling. He remains aphasic, albeit with good understanding (and can use pictograms and gestures to communicate), and with bilateral spasticity and dystonia and limited walking range. There have been no further episodes of decompensation up to the age of 12 years. His head circumference is on the 25^th^ centile.

His father was clinically asymptomatic at the age of 45 years

ISGs were tested on a single occasion, with an interferon score of 7.899 at age 6.79 years. Paternal ISGs were normal when tested at the age of 45 years.

Sequencing identified a paternally inherited heterozygous c.590A>G (p.(Lys197Arg)) (K197R) variant in *PTPN1* (the same variant seen in his affected cousin AGS492.1), which was not present on gnomAD v4.1.0.

**AGS761**. Male born with normal weight (> 7 lb) to non-consanguineous parents after an unremarkable pregnancy and neonatal period. Having followed a completely normal developmental trajectory, a week following scheduled 15-month vaccinations, he presented with fever and irritability, accompanied by a rapid loss of motor skills, language, the ability to self-feed and to swallow (requiring the placement of a gastrostomy tube), with elevated liver enzymes and raised CSF neopterin. Cerebral imaging at age 16 months revealed patchy high T2 signal white matter abnormalities and cerebral atrophy. No information on subsequent neuroimaging is available. He was treated with intravenous immunoglobulin (IVIG) (once per 3 – 4 weekly for 6 months) without obvious benefit. Characterised by truncal hypotonia and spastic-dystonic quadriparesis, by age 2 years his condition appeared to have stabilised, and there have been no further episodes of decompensation up to the age of 12 years. He is wheelchair-bound, cannot feed himself, sit, stand or even roll over.

CSF neopterin was 300 nmol/L at age 16 months, and 239 nmol/L at age 21 months (normal range 7 – 65 nmol/L). Interferon alpha activity in the CSF was elevated at 18 IU/mL and 9 IU/mL (normal < 3 IU/mL) at 18 and 20 months of age respectively. ISGs tested on two occasions were also elevated, with interferon scores of 7.8 and 4.683 at ages 1.51 and 1.69 years respectively. Maternal and paternal ISGs were normal on both occasions tested.

Sequencing identified a de novo heterozygous c.63+1G>C variant (paternity confirmed) in *PTPN1*, which was not present on gnomAD v4.1.0.

**AGS1036** (See video 2, Case 2 in Sa et al.^10^). A previously well 5-year-old girl with a normal antenatal and developmental profile, presented with a subacute onset over three months of left sided weakness, frequent falls and difficulties climbing stairs. She was also unable to raise her left arm and had problems dressing and undressing. Her right side was also affected and her handwriting deteriorated. Her speech deteriorated and she developed swallowing difficulties with excessive drooling. There was no cognitive regression, sleep disturbance or history of seizures. On examination, her speech was dysarthric. She had a mixture of spasticity and dystonia with lower limbs more affected than upper limbs and left side more than right. Her tone was increased bilaterally with sustained clonus on the left and upgoing plantars bilaterally. Head circumference was normal. Brain MRI at age 5 years (5 months after symptom onset) demonstrated generalized volume loss more marked in the right cerebral hemisphere. Brain biopsy showed a chronic inflammatory infiltrate (T-cells and microglia, with tubuloreticular inclusions - a marker of interferon signalling in the endothelium.^11^). Liver transaminases were elevated (ALT maximum of 393 U/L). At 12 months from onset the patient was treated with four cycles of pulse intravenous (IV) methylprednisone (four cycles of 30 mg/kg/d for 5 days 3 – 4 months apart) and IVIG (2 g/kg) three to four months apart. This resulted in significant improvement in her movement disorder and in all domains of function (gross motor, fine motor and speech). She returned almost back to normal with only some residual lower limb hypertonia. Further imaging 23 months after treatment (and 35 months from symptom onset) showed significant reversal of the previously noted volume loss.

Her mother is clinically asymptomatic at the age of 37 years.

CSF neopterin was elevated (200 nmol/L: normal range 7 – 65 nmol/L) at the age of 5 years, and had fallen to within the normal range 24 months thereafter (29 nmol/L). ISGs were initially elevated on four occasions (interferon scores of 8.042, 8.447, 9.751 and 8.297 at ages 5.24, 5.32, 5.39 and 5.75 years respectively), falling to normal thereafter (interferon scores of 1.953, 1.348 and 0.663 at ages 6.05, 6.11 and 6.82 respectively). Maternal and paternal ISGs were not tested.

Sequencing identified a maternally inherited heterozygous c.466C>T (p.(Arg156*)) (R156*) variant in *PTPN1* not present on gnomAD v4.1.0 (previously reported in primary mediastinal B cell lymphoma by Gunawardana et al. 2014.^12^). This variant was also observed to have arisen de novo in AGS2942. A younger brother (mentioned in Sa et al.^10^) did not carry this variation.

**AGS1312** (See video 3, Case 3 in Sa et al.^10^). A previously well 4-year-old girl with mild gross motor developmental delay (walking independently at age 21 months), presented with a progressive one-year history of frequent trips and falls, difficulty climbing stairs, and progressive walking difficulty needing the aid of a wheelchair for outdoors. There was also five-month history of speech regression; initially slurred incomprehensible words, followed by complete loss of expressive language. She had dysphagia with choking on solids and liquids and drooling. Behavioural difficulties were also reported with frequent angry outbursts and peer-relationship problems. There was no history of seizures. On examination, her tone was increased in the lower limbs with mixed spasticity and dystonia (right worse than left), and bilateral upgoing plantars. She walked with a wide gait with both her knees hyperextended. There was drooling but no cranial nerve abnormalities. Head circumference was below the 0.4th centile (no previous measurements available for comparison). There was no hepatosplenomegaly. MRI brain at age 4 years (9 months after symptom onset) showed mild global cerebral volume loss mimicking atrophy. Liver transaminases were minimally elevated (ALT maximum of 37 U/L). At 15 months from onset she had one course of three days IV methylprednisolone (30 mg/kg/d), followed by IVIG (2 g/kg) cycles, five courses over 20 months. Marked improvement in her lower limb tone, with gain in both motor and expressive language skills, was observed. A repeat MRI at 13 months after treatment initiation (and 30 months after symptom onset) showed reversal of previous changes. Currently, she is independently ambulant, and able to climb stairs unaided, although she still has a spastic paraplegia. Her speech and swallowing have returned to normal.

Her father is clinically asymptomatic at the age of 48 years.

CSF neopterin was elevated (151 nmol/L, and 93 nmol/L: normal range 7 – 65 nmol/L) at the age of 5 years and 12 months thereafter. ISGs tested on four occasions were elevated, with interferon scores of 4.913, 4.45, 5.312 and 3.905 at ages 4.6, 4.97, 5.29 and 5.56 years respectively. Maternal and paternal ISGs were normal on both occasions tested.

Sequencing identified a paternally inherited heterozygous c.1000delG (p.(Val334*)) (V334*) variant in *PTPN1* not present on gnomAD v4.1.0.

**AGS1421** (See video 1, Case 1 in Sa et al.^10^). This previously developmentally normal male presented at age 22 months with a progressive left-sided hemiparesis. Five months later, he experienced fever, and the evolution of additional right sided weakness, together with speech regression and swallowing difficulties. Neurological examination revealed a spastic-dystonia more marked in the lower limbs. Head circumference was normal. ~~C~~erebral MRI at around 27 months of age demonstrated initially right hemispheric atrophy, subsequently evolving to global symmetric cerebral volume loss at age 34 months (12 months after symptom onset). Liver transaminases were elevated (ALT maximum of 90 U/L). Brain biopsy revealed patchy gliosis with good neuronal preservation and no significant inflammation. 18 months after the initial presentation he was started on 6 weekly pulses of IV methylprednisolone (30 mg/kg/d), followed by six weekly IVIG (2 g/kg). Treatment was associated with an improvement in fine motor and non-verbal communication skills, and less so in gross motor skills and speech. Aged 10 years his condition is stable, being able to sit unsupported, with the continued acquisition of communication skills. Repeat brain MRI 26 months after treatment (and 44 months after symptom onset) showed complete reversal of previous changes. There have been no further episodes of decompensation up to the age of 11 years.

His father is clinically asymptomatic at the age of 49 years.

CSF neopterin was elevated (355 nmol/L, normal range 7 – 65 nmol/L) at age 22 months. ISGs tested at age 3.26 years were significantly elevated (interferon score of 16.135), but had normalised when reassessed at age 4.66 years.

Sequencing identified a paternally inherited heterozygous c.370_386del (p.(Tyr124Argfs*7)) (Y124Rfs*7) variant in *PTPN1* not present on gnomAD v4.1.0.

**AGS2942**. This female child, born at term with a normal birth weight and head circumference to non-consanguineous parents, demonstrated completely normal psychomotor development until 8 years of age. Then, over a period of approximately 1 year, she developed a left sided limp when running associated with a degree of lower limb spasticity, and began to demonstrate clear cognitive decline with reduced school performance, slowed dressing, asthenia, speech difficulties, a deterioration in her writing, disturbed sleep and recurrent fevers progressing over a period of one year. At this age (8 years), there was definite cerebral atrophy and some T2 Flair hypersignal of the deep white matter of the corona radiata were seen on MRI, with an absence of calcification on cerebral CT. An EEG was normal. Now age 11 years, her condition has stabilised and she has regained skills, so that she no longer has features of lower limb spasticity, being able to walk and run without problem. She has continued slight difficulties with articulation. Vision and hearing are normal. She has experienced no further fevers. She has started puberty. Her most recent cerebral MRI at age 10 years 5 months showed a resolution of the features of cerebral atrophy seen previously, and a reduction of the T2 high signal white matter changes.

CSF neopterin (1100 nmol/L, range 10 – 24 nmol/L), as well as interferon alpha protein in the CSF (5706 fg/mL: normal <10 fg/mL) and serum (1448 and 1302 fg/mL: normal <10 fg/mL) were grossly elevated at age 10 years. ISGs tested on two occasions revealed interferon scores of 4.535 and 2.985 at 10.11 and 10.48 years of age respectively.

Sequencing identified a de novo heterozygous c.466C>T (p.(Arg156*)) (R156*) variant in *PTPN1* not present on gnomAD v4.1.0 (previously reported in primary mediastinal B cell lymphoma by Gunawardana et al.^12^). This variant was also observed in AGS1036 where it was inherited from an asymptomatic mother.

**AGS3148**. This male exhibited completely normal development during infancy, walking independently at the age of 10 months, and speaking his first words at 12 months of age. Aged 15 months he experienced a febrile seizure, and one month later he was noted to be losing skills, so that by age 19 months his speech was completely absent, he was unable to walk, and he demonstrated upper and lower limb spasticity. Cranial MRI at age 16 months showed increased T2 signal white matter in a periventricular and frontotemporal distribution consistent with a leukodystrophy. Follow-up brain imaging at age 33 months showed an improvement in the white matter signal, which had almost normalised on repeat imaging at age 68 months, and remained stable when last imaged at age 14 years.. At age 13 years his head circumference was 55cm (25-50^th^ centile). Now, aged 17 years, he demonstrates profound developmental delay with severe spasticity of upper and lower limbs with flexion contractures, and features of pseudobulbar syndrome.

ISGs tested on one occasion revealed an interferon score of 5.2 at age 17.79 years.

Sequencing identified an apparently de novo heterozygous c.154+1del variant in *PTPN1* not present on gnomAD v4.1.0.

**AGS3165**. This male demonstrated normal development until age 4.5 years (54 months), at which time he experienced a febrile episode associated with irritability and the onset of spasticity, dysarthria and swallowing difficulties progressing rapidly over 2 months, losing the ability to feed and dress himself, and to climb stairs. There was progression of his disease over a few months so that by age 5 years he was unable to walk, his language had become incomprehensible, and his swallowing of liquids unsafe. He was treated at age 58 months with 3 doses of corticosteroid without obvious benefit. Aged 63 months steroids (1mg/kg/day) were restarted, followed by a definite improvement in walking and swallowing. By age 6 years he was regaining developmental skills, albeit in the context of a significant spastic paraparesis, becoming able to feed himself again, and demonstrating language skills appropriate for his age. Initial cerebral imaging (MRI and CT) at age 54 months was normal, but subsequent neuroimaging at age 58 months demonstrated confluent T2 and FLAIR high signal involving the frontal supratentorial juxta-ventricular white matter bilaterally but with right subcortical predominance with no associated diffusion abnormality. Cerebral CT did not reveal any calcifications. Repeat cerebral MRI at age 61 months showed almost complete resolution of the white matter abnormalities previously seen.

His mother is clinically asymptomatic at the age of 34 years.

CSF neopterin was elevated at 323 nmol/L and 162 nmol/L (normal range 7 – 55 nmol/L) aged 5 and 6 years respectively. ISGs tested on two occasions revealed interferon scores of 7.29 and 5.925 (at ages 4.85 and 5.46 years respectively). ISGs tested in the clinically asymptomatic mother were normal (interferon score of 1.29) at age 33 years.

Sequencing identified a maternally inherited heterozygous c.784C>T (p.(Gln262*)) (Q262*) variant in *PTPN1* not present on gnomAD v4.1.0.

**AGS3479**. This female was born to non-consanguineous parents and demonstrated a normal developmental profile up to 1.5 years of age. At this time she developed a left-sided hemiparesis with marked contralateral hemispheric atrophy on MRI at age 19 months. There was no associated white matter disease. Brain biopsy at the age of 2 years and 3 months showed areas of gliosis, focal perivascular lymphohistiocytic infiltrates and suspected partial myelin loss. Over a several month period her disease evolved to four-limb spastic-dystonia with drooling, dysphagia and no speech. Exhaustive workup was notable only for raised CSF neopterin (330 nmol/L, normal <30 nmol/L). A second MRI at age 22 months showed extension of the atrophy to the other hemisphere. No further imaging has been undertaken. Treatment with IVIG was initiated at age 23 months, but stopped shortly thereafter because of an allergic reaction. Now, at the age of 4 years 5 months, she is completely bed-ridden, with four-limb spastic-dystonia and no language although she can manifest a social smile.

Her mother is clinically asymptomatic at the age of 43 years.

ISGs tested on three occasions revealed interferon scores of 8.01, 8.67 and 7.52 at the ages of 2.46, 2.66 and 4.06 years respectively. ISGs tested in the clinically asymptomatic mother were minimally elevated (interferon score of 2.91) at age 43 years.

Sequencing identified a maternally inherited heterozygous c.166C>T (p.(Arg56Trp)) (R56W) variant in *PTPN1* not present on gnomAD v4.1.0 (previously reported in Hodgkin lymphoma by Gunawardana et al.^12^).

**AGS3542**. Following completely normal development, this female presented at age 23 months with progressive spastic tetraparesis more pronounced on the left side (although, in retrospect, a problem with left hand grasping might have been present since age 4 months), with initially normal cognition and speech. She experienced a further period of regression at age 36 months which also affected her speech and swallowing, resulting in limited articulation and drooling. Cerebral MRI at age 26 months demonstrated right sided hemiatrophy which had extended bilaterally by the age of 44 months. There was significant clinical improvement, following pulsed methylprednisolone first started at age 27 months for 3 months (20mg/kg on 5 consecutive days in each of 3 months), with a further 4 weeks of pulsed methylprednisolone (20mg/kg on 3 consecutive days weekly) following a relapse in her neurological state at age 38 months. Together with mycophenolate (900 mg/m^2^/day, then 600 mg/m^2^/day), methylprednisolone was reinitiated at age 51 months because of further slow regression. Mycophenolate was stopped after 15 months and the child remained neurologically dependent on steroids (20mg/kg on 3 consecutive days repeated at 4 week intervals over a several month period), with further regression if the off-treatment period was extended beyond 4 weeks. Steroids were replaced by immunoglobulins at age 7 years for some months, and she remained stable with some developmental progress. Repeat brain imaging at age 60 months showed that brain volume had almost normalised, and has remained unchanged since then (with the last cerebral imaging at age 92 months. Now, she has only very few words (< 20), but has relatively well-preserved communication via a computer-assisted language tool, and she can use an electric wheelchair unaided.

Her father is clinically asymptomatic at the age of 46 years.

CSF neopterin was elevated at 114 nmol/L and 352 nmol/L (range 5 – 53 nmol/L) at the time of her first regression at age 2 years, 352 nmol/L during her more severe episode of regression at age 3 years respectively, and was normal when tested at 7 years of age at which time her disease had apparently stabilised and she was making developmental progress. ISGs in blood were elevated at the age 8.22 years, with an interferon score of 3.105. ISGs tested in the clinically asymptomatic father were normal (interferon score of -1.28) at age 46 years.

Sequencing identified a paternally inherited heterozygous c.505C>T (p.(Arg169*)) (R169*) variant in *PTPN1* not present on gnomAD v4.1.0.

**AGS3561**. This female is the youngest of 8 children born to a non-consanguineous couple. After normal development, shortly following infection with SARS-CoV-2, at age 7 years she experienced neurological regression with a progressive gait abnormality, speech disturbance and mild cognitive decline. She began to walk slowly, with unstable gait and recurrent falls, demonstrating cerebellar, extrapyramidal and pyramidal signs, weakness and dystonic posturing more so on the right side of the body. Her speech was dysarthric, so that she could only manage simple and short conversations, and her eating was slow and disorganized, albeit without aspiration. CSF neopterin was measured at 139 nmol/L at the age of 8 years (normal range < 20 nmol/L). There has been a subsequent re-attainment of some skills, so that now at the age of 120 months she speaks in relatively simple sentences with reduced comprehension. She can walk independently but only over a short distance. Cerebral MRI at age 92 months showed diffuse, patchy high T2 white matter signal abnormalities with cerebral atrophy. The cerebral atrophy was more obvious when repeated at age 96 months. No further imaging has been undertaken. Her head circumference at age 95 months was 54 cm (98 percentile; in line with parental head size).

Her father is clinically asymptomatic at the age of 52 years.

ISGs tested on one occasion revealed an interferon score of 7.27 at age 8.28 years.

Sequencing identified a paternally inherited heterozygous c.619G>T (p.(Glu207*)) (E207*) variant in *PTPN1* not present on gnomAD v4.1.0.

**SUPPLEMENTARY FIGURES**

**Supplementary Figure 1**. **Evidence of upregulated type I interferon signalling in patients**

(A) Clustal Omega alignment of PTP1B, with the two non-synonymous missense substitutions identified in our cohort highlighted in yellow. Alignments are based on the human transcript of *PTPN1* / PTP1B: ENST00000371621.5; NM_002827.4.; NP_002818.1. (B) Details of interferon (IFN) scores (median fold expression of a panel of IFN stimulated genes (ISGs)) recorded in symptomatic patients carrying a mutation in *PTPN1,* as in Figure 3, with patients represented by different colours, age at sampling in years represented on the X-axis and scores obtained after treatment initiation represented by crossed circles. IFN scores were derived using the six ISG panel measured by qPCR (left), or the 24 ISG panel generated using NanoString technology (right). The upper range of normal is calculated as +2 SD above the mean of the control group, as indicated by the dotted lines: qPCR = 2.466; NanoString = 2.758. (C) Tubuloreticular inclusions in the brain biopsy of AGS1036. Ultrastructural examination of cerebral white matter of AGS1036 taken at age five years reveals clusters of tubuloreticular inclusions (white arrows), in a blood vessel endothelial cell. Original magnification x 6,000.^13^

**Supplementary Figure 2. Sashimi plots**

(A) Sashimi plots showing exon–exon junctions and splicing for *PTPN1* transcripts obtained by next generation sequencing of captured *PTPN1* cDNA from control (HDF) and patient AGS3148 primary fibroblast RNA. The *PTPN1* gene locus is shown along the horizontal axis. Histogram spikes represent the amount of reads sequenced for the relevant location, and total ranges of read number are indicted on the left. Numbers over the lines connecting exons represent the number of reads mapped to relevant exon–exon junctions: bold indicates a change in abundance, and blue colour indicate a novel junction. (B) Zoom of frame in Sashimi plot in (A): appearance of a new splicing site inside exon 2. (C) Sashimi plots showing exon–exon junctions and splicing for *PTPN1* transcripts obtained by next generation sequencing of captured *PTPN1* cDNA from control (Ctrl) and AGS761 patient whole blood RNA. The *PTPN1* gene locus is shown along the horizontal axis. Histogram spikes represent the amount of reads sequenced for the relevant location, and total ranges of read number are indicted on the left. Numbers over the lines connecting exons represent the number of reads mapped to relevant exon–exon junctions: bold indicates a change in abundance, and green colour indicate a novel junction. (D) Zoom of frame in Sashimi plot in (C): appearance of a new splicing site (green arrow) in intron 1 leading to intron retention. (E) Coverage of *PTPN1* exon 1 and the beginning of intron 1 showing partial intron 1 retention (green arrow shows the new splicing site) and base prevalence in reads: the mutated allele C is predominant in this mRNA species with intron retention. (F) Schematic representation of full-length cDNA of WT and mutated *PTPN1.* The coding sequences of the exons are numbered and shown in grey, and the 5′- or 3′-UTR is shown in smaller open boxes. For the c.154+1del variant, capture RNA sequencing showed transcripts with complete skipping of exon 2 skipping, and others in which an alternative splice site in exon 2 was used, represented by hashed blue boxes leading to stop codons, symbolised by an open box downstream. For the c.63+1G>C variant, we found a minor cDNA species with partial intron 1 retention, represented by the green box, leading to a stop codon in exon 3.

**Supplementary Figure 3**. **Characterisation of PTP1B WT and KO BJ-5ta human fibroblast cell clones and global phospho-tyrosine levels**

(A) Western blot analysis of PTP1B expression in BJ-5ta human fibroblast cell lines either wild-type (WT) or homozygous knockout (KO) for PTP1B generated using CRISPR-Cas9 gene editing. Vinculin is used as a loading control. (B) GSEA (gene set enrichment analysis) bar plot displaying up- and down-regulated pathways in *PTPN1* KO versus WT clones at baseline in bulk RNA sequencing of BJ-5ta human fibroblast clones. Differentially expressed genes (DEGs) with an adjusted *p* value < 0.1 were analysed. Enriched pathways with a nominal *p* value < 0.05 and FDR (false discovery rate) < 0.1 are shown. NES, normalised enrichment score. (C) Western blot analysis of PTP1B expression in BJ-5ta human fibroblast cell lines either wild-type (WT), homozygous knockout (KO), heterozygous KO (WT/KO) or heterozygous knock-in (KI) for c.63+1G>C (WT/KI) for *PTPN1* generated using CRISPR-Cas9 gene editing. Vinculin is used as a loading control. (D) Cartoon depicting the role of PTP1B in STING and IFNAR signalling pathways (created with BioRender.com). (E - F) Representative immunoblots of total phospho-tyrosine (p-Tyr) protein levels in a time-course experiment following stimulation with 1000 IU/mL IFNα2b (E) or 1 µM diABZI (F) for the indicated times. WT, one clone; KO, one clone. UT, untreated. Perpendicular scales (0 to 400) on the left indicate the direction and distance in the blots for densitometry quantification in G, H and I. Purple stars, triangles and rectangles indicate the lanes used for densitometry quantification in G, H and I, respectively. Note the transposition of the 4h and 2h samples for the KO clone in (E). (G- I) Densitometry quantification of lanes in E and F, as described above, for total phospho-tyrosine protein levels at baseline (G), and following stimulation with IFNα2b (H) for 1 hour, and with diABZI (I) for 4 hours. AUC, area under curve.

**Supplementary Figure 4**. **IFN signalling upon stimulation in PTP1B WT and KO BJ-5ta human fibroblast cell clones**

(A-C) qPCR analysis of ISG expression following stimulation with either 200 IU/mL IFNα2b (A) or 0.01 µM diABZI (B) for 6 hours in WT, KO, WT/KO and WT/KI BJ-5ta human fibroblast cell clones. (C) *IFNB1* mRNA expression in WT, KO, WT/KO and WT/KI BJ-5ta human fibroblast cell clones stimulated by diABZI. Each circle or triangle represents the average ISG score or *IFNB1* expression from independent clones of the same genotype (WT, two to four clones; WT/KO, four clones; WT/KI, one clone) in one experiment. ISG score is calculated as above. Ratio paired *t* test was used to compare the ISG or *IFNB1* expression levels in WT and mutant BJ-5ta clones following stimulation with IFNα2b (A) or diABZI (B-C). (D-E) Representative immunoblots of time-course stimulation with 1000 IU/mL IFNα2b (D) or 1 µM diABZI (E) for the indicated times. Arrow in E indicates the band for total STING. The band above it is p-STING. Quantification of p-STAT1 (1 h IFNα2b), p-STING (4 h diABZI) and STING (UT) relative to Vinculin is given to the right of the relevant blot. Unpaired *t* test was used to compare WT and KO BJ-5ta clones. WT, one clone; KO, one clone. UT, untreated.

**Supplementary Figure 5. Characterisation of *PTPN1* KO hTERT RPE-1 cells**

(A) Representative immunoblot showing baseline PTP1B expression in CRISPR-engineered hTERT RPE-1 PTP1B wild-type (WT) and knock-out (KO) pools. (B) qPCR of baseline expression of ISGs in *PTPN1* WT and KO hTERT RPE-1 cells. Unpaired t test was used to compare the levels of baseline ISG expression in WT and KO cells. The ISG score was calculated as the median of the expression of four ISGs*, IFI27*, *IFI16*, *MX1* and *IRF7*. (C) Representative immunoblots demonstrating KO of *STING1* and *IFNAR1* in *PTPN1* KO cells. For the IFNAR1 blot, samples were treated with PNGase F to remove glycosylation prior to gel electrophoresis.

**SUPPLEMENTARY TABLES**

**SUPPLEMENTARY TABLES**

**Supplementary Table 1. Summary data of antibody testing, brain biopsy, miscellaneous laboratory investigations and temporal relationship of onset to vaccination by patient**

| **Family** | **Neural antibodies*** | **Systemic antibodies** | **Brain biopsy** | **Other** | **Temporal relation to vaccination** |
| --- | --- | --- | --- | --- | --- |
| **AGS492.1** | NA | NA | No |  | Not known |
| **AGS492.4** | Negative | ANA 1/320; anti-dsDNA, ANCA, antiphospholipid, anti-thyroglobulin negative | Perivascular inflammation in cortex, WM and meninges with minimal CD68 microglial nodules/CD3 and CD20 lymphocytes. Structural disorganisation with ectopic neurons and florid gliosis in WM | Non-specific creatine and inositol peaks on MRS. Normal CSF cells, protein, glucose. Extensive CSF infection screen negative. Lymphocyte phenotyping normal. Modest elevation of C26:C22 ratio | No |
| **AGS761** | NA | NA | No |  | Onset within 3 weeks of DTaP-IPV/Hib vaccine |
| **AGS1036 (Case 2 in Sa et al.)^10^** | Negative | NA | T-cells (CD3) and microglia (CD68) inflammatory cells, with endothelial tubuloreticular inclusions | Normal CSF cells and protein. No oligoclonal bands. Deep RNA sequencing for viruses in brain biopsy normal | No |
| **AGS1312 (Case 3 in Sa et al.)^10^** | Negative | NA | No | Normal CSF cells and protein. No oligoclonal bands | No |
| **AGS1421 (Case 1 in Sa et al.)^10^** | Negative | NA | Patchy gliosis only with no features of inflammation | Normal CSF cells and protein. No oligoclonal bands. Deep RNA sequencing for viruses in brain biopsy normal | No |
| **AGS2942** | NA | Negative | No | No T, B or NK lymphopenia. Decrease of TEMRA CD8+ T cells (CD45RA+CCR7-), moderate excess of memory CD8+ T cells (CD45RA-CCR7-) = non-specific abnormalities Minor elevation of C26:0/C22 ratio | No |
| **AGS3148** | NA | NA | No |  | Not known |
| **AGS3165** | Negative | Negative | No |  | No |
| **AGS3479** | NA | ANA, anti-dsDNA negative | Areas of gliosis, focal perivascular lymphohistiocytic infiltrates and suspected partial myelin loss |  | No |
| **AGS3542** | Negative | Minimally positive ANA on 2 occasions; RhF, anti-dsDNA negative | No | OGB seen; CSF glucose, cells, protein, lactate negative | No |
| **AGS3561** | Negative | NA | No | Normal CSF cells, glucose and protein; MRS normal | No (but onset of neurological disease appeared to coincide with SARS-CoV-2 infection) |

*Determined using live cell-based assays

ANA: anti-nuclear antibodies; ANCA: anti-neutrophil cytoplasmic antibodies; CSF: cerebrospinal fluid; IVIG: intravenous immunoglobulin; IV: intravenous; MRS: magnetic resonance spectroscopy; NA: not assessed; OGB: oligoclonal bands; RhF: rheumatoid factor antibodies; WM = white matter

**Supplementary Table 2. Interferon signalling status and CSF pterins by patient**

| **Family** | **IS (age in years)*** | **IFN-alpha protein ** (age)** | **IFN-alpha activity (age)** | **CSF pterins *** (age)** |
| --- | --- | --- | --- | --- |
| **AGS492.1** | 8.131 (3.62); 8.009 (4.1) | NA | CSF 6IUm/l (2.5y); serum 9IU/ml (2.5y) | >2 (2.5y) |
| **AGS492.4** | 7.899 (6.79) | NA | NA | NA |
| **AGS761** | 7.818 (1.51); 4.683 (1.69) | NA | CSF 18IU/ml (18m); CSF 9IU/ml (20m) | >10 (16m); >4 (16m); >3 (21m) |
| **AGS1036 (Case 2 in Sa et al.)^10^** | 8.042 (5.24); 8.447 (5.32); 9.751 (5.39); 8.297 (5.75); 1.953 (6.05); 1.348 (6.11); 0.663 (6.82) | NA | NA | >3 (5y); Normal (7y) |
| **AGS1312 (Case 3 in Sa et al.)^10^** | 4.913 (4.6); 4.45 (4.97); 5.312 (5.29); 3.905 (5.56) | NA | NA | >2 (5y); 1.5 (6y) |
| **AGS1421 (Case 1 in Sa et al.)^10^** | 16.135 (3.26); 1.112 (4.66) | NA | NA | >5 (22m) |
| **AGS2942** | 4.535 (10.11); 2.985 (10.48); 1.935 (10.95) | CSF 5706 (10y); serum: 1448 (10y 5m) and 1302 (10y 11m) | NA | >45 (10y) |
| **AGS3148** | 5.2 (17.79) | NA | NA | NA |
| **AGS3165** | 7.29 (4.85); 5.925 (5.46) | NA | NA | >5 (5y); ~3 (6y) |
| **AGS3479** | 8.01 (2.46); 8.67 (2.66)  7.52 (4.06) | NA | NA | >10 (2y) |
| **AGS3542** | 3.105 (8.22) | NA | NA | >2 (2y); >6 (3y); Normal (7y) |
| **AGS3561** | 7.27 (8.28) | NA | NA | >7 (8y) |

CSF = cerebrospinal fluid; NA = not assessed; IFN = interferon; IS = interferon score; IU = international units; m = months; y = years

* First number = IS; number in brackets = age at sampling in decimalised years. IS generated using RT-qPCR in families AGS492 – AGS1421 (normal < 2.46); IS generated by NanoString in all other families (Normal < 2.75)

** Normal in serum and CSF < 10 fg/mL

*** First number = Number of multiples above the upper limit of normal; number in brackets = age at sampling

**SUPPLEMENTARY REFERENCES**

1. Sobreira N, Schiettecatte F, Valle D, Hamosh A. GeneMatcher: a matching tool for connecting investigators with an interest in the same gene. *Hum Mutat* 2015; **36**(10): 928-30.

2. Palisano R, Rosenbaum P, Walter S, Russell D, Wood E, Galuppi B. Development and reliability of a system to classify gross motor function in children with cerebral palsy. *Dev Med Child Neurol* 1997; **39**(4): 214-23.

3. Hidecker MJ, Paneth N, Rosenbaum PL, et al. Developing and validating the Communication Function Classification System for individuals with cerebral palsy. *Dev Med Child Neurol* 2011; **53**(8): 704-10.

4. Sharma S, Skaist Mehlman T, Sagabala RS, Boivin B, Keedy DA. High-resolution double vision of the allosteric phosphatase PTP1B. *Acta Crystallogr F Struct Biol Commun* 2024; **80**(Pt 1): 1-12.

5. Pettersen EF, Goddard TD, Huang CC, et al. UCSF ChimeraX: Structure visualization for researchers, educators, and developers. *Protein Sci* 2021; **30**(1): 70-82.

6. Guerois R, Nielsen JE, Serrano L. Predicting changes in the stability of proteins and protein complexes: a study of more than 1000 mutations. *J Mol Biol* 2002; **320**(2): 369-87.

7. Rice GI, Forte GM, Szynkiewicz M, et al. Assessment of interferon-related biomarkers in Aicardi-Goutieres syndrome associated with mutations in TREX1, RNASEH2A, RNASEH2B, RNASEH2C, SAMHD1, and ADAR: a case-control study. *Lancet Neurol* 2013; **12**(12): 1159-69.

8. Lepelley A, Martin-Niclos MJ, Le Bihan M, et al. Mutations in COPA lead to abnormal trafficking of STING to the Golgi and interferon signaling. *J Exp Med* 2020; **217**(11).

9. Subramanian A, Tamayo P, Mootha VK, et al. Gene set enrichment analysis: a knowledge-based approach for interpreting genome-wide expression profiles. *Proc Natl Acad Sci U S A* 2005; **102**(43): 15545-50.

10. Sa M, Hacohen Y, Alderson L, et al. Immunotherapy-responsive childhood neurodegeneration with systemic and central nervous system inflammation. *Eur J Paediatr Neuro* 2018; **22**(5): 882-8.

11. Rich SA. Human lupus inclusions and interferon. *Science* 1981; **213**(4509): 772-5.

12. Gunawardana J, Chan FC, Telenius A, et al. Recurrent somatic mutations of PTPN1 in primary mediastinal B cell lymphoma and Hodgkin lymphoma. *Nat Genet* 2014; **46**(4): 329-35.

13. Elmaghrabi A, Brown E, Khin E, Hassler J, Hendricks AR. Tubuloreticular Inclusions in the Absence of Systemic Lupus Erythematosus and HIV Infection: A Report of Three Pediatric Cases. *Case Rep Nephrol Dial* 2017; **7**(2): 91-101.
